# Investigating the Paracrine Role of Perinatal Derivatives: Human Amniotic Fluid Stem Cell-Extracellular Vesicles Show Promising Transient Potential for Cardiomyocyte Renewal

**DOI:** 10.3389/fbioe.2022.902038

**Published:** 2022-06-08

**Authors:** Ambra Costa, Carolina Balbi, Patrizia Garbati, Maria Elisabetta Federica Palamà, Daniele Reverberi, Antonella De Palma, Rossana Rossi, Dario Paladini, Domenico Coviello, Pierangela De Biasio, Davide Ceresa, Paolo Malatesta, Pierluigi Mauri, Rodolfo Quarto, Chiara Gentili, Lucio Barile, Sveva Bollini

**Affiliations:** ^1^ Experimental Biology Unit, Department of Experimental Medicine (DIMES), University of Genova, Genova, Italy; ^2^ Laboratory of Cellular and Molecular Cardiology, Istituto Cardiocentro Ticino, Ente Ospedaliero Cantonale, Lugano, Switzerland; ^3^ Center for Molecular Cardiology, University of Zurich, Zurich, Switzerland; ^4^ Laboratories for Translational Research, Ente Ospedaliero Cantonale, Bellinzona, Switzerland; ^5^ Molecular Pathology Unit, IRCCS Ospedale Policlinico San Martino, Genova, Italy; ^6^ Proteomics and Metabolomics Unit, Institute for Biomedical Technologies (ITB-CNR), Milan, Italy; ^7^ Fetal Medicine and Surgery Unit, IRCCS Istituto Giannina Gaslini, Genova, Italy; ^8^ Laboratory of Human Genetics, IRCCS Istituto Giannina Gaslini, Genova, Italy; ^9^ Prenatal Diagnosis Perinatal Medicine Unit, IRCCS Ospedale Policlinico San Martino, Genova, Italy; ^10^ Cellular Oncology Unit, IRCCS Ospedale Policlinico San Martino, Genova, Italy; ^11^ Laboratory for Cardiovascular Theranostics, Istituto Cardiocentro Ticino, Ente Ospedaliero Cantonale, Lugano, Switzerland; ^12^ Faculty of Biomedical Sciences, Università Svizzera Italiana, Lugano, Switzerland

**Keywords:** perinatal derivatives, human amniotic fluid stem cells, extracellular vesicles, secretome, paracrine effects, myocardial renewal, regenerative medicine

## Abstract

Cardiomyocyte renewal represents an unmet clinical need for cardiac regeneration. Stem cell paracrine therapy has attracted increasing attention to resurge rescue mechanisms within the heart. We previously characterized the paracrine effects that human amniotic fluid–derived stem cells (hAFSC) can exert to provide cardioprotection and enhance cardiac repair in preclinical models of myocardial ischemia and cardiotoxicity. Here, we analyze whether hAFSC secretome formulations, namely, hAFSC conditioned medium (hAFSC-CM) over extracellular vesicles (hAFSC-EVs) separated from it, can induce cardiomyocyte renewal. c-KIT+ hAFSC were obtained by leftover samples of II trimester prenatal amniocentesis (fetal hAFSC) and from clinical waste III trimester amniotic fluid during scheduled C-section procedures (perinatal hAFSC). hAFSC were primed under 1% O_2_ to enrich hAFSC-CM and EVs with cardioactive factors. Neonatal mouse ventricular cardiomyocytes (mNVCM) were isolated from cardiac tissue of R26pFUCCI2 mice with cell cycle fluorescent tagging by mutually exclusive nuclear signal. mNVCM were stimulated by fetal versus perinatal hAFSC-CM and hAFSC-EVs to identify the most promising formulation for *in vivo* assessment in a R26pFUCCI2 neonatal mouse model of myocardial infarction (MI) *via* intraperitoneal delivery. While the perinatal hAFSC secretome did not provide any significant cardiogenic effect, fetal hAFSC-EVs significantly sustained mNVCM transition from S to M phase by 2-fold, while triggering cytokinesis by 4.5-fold over vehicle-treated cells. Treated mNVCM showed disorganized expression of cardiac alpha-actinin, suggesting cytoskeletal re-arrangements prior to cell renewal, with a 40% significant downregulation of *Cofilin-2* and a positive trend of polymerized F-Actin. Fetal hAFSC-EVs increased cardiomyocyte cell cycle progression by 1.8-fold in the 4-day-old neonatal left ventricle myocardium short term after MI; however, such effect was lost at the later stage. Fetal hAFSC-EVs were enriched with a short isoform of Agrin, a mediator of neonatal heart regeneration acting by YAP-related signaling; yet *in vitro* application of YAP inhibitor verteporfin partially affected EV paracrine stimulation on mNVCM. EVs secreted by developmentally juvenile fetal hAFSC can support cardiomyocyte renewal to some extension, *via* intercellular conveyance of candidates possibly involving Agrin in combination with other factors. These perinatal derivative promising cardiogenic effects need further investigation to define their specific mechanism of action and enhance their potential translation into therapeutic opportunity.

## 1 Introduction

Cardiac disease can arise from alterations in the heart structure and function, such as myocardial infarction (MI), chemotherapy-derived cardiotoxicity, and congenital heart defects, all progressively leading to heart failure. The main challenge related to MI is the loss of functional contractile cardiomyocytes during a prolonged ischemic insult. Regenerative therapies aiming at promoting cell cycle re-entry and proliferative activity of resident cardiomyocytes have been broadly explored to overcome the challenge of defective myocardial repair following injury. Targeting myocardial renewal to enhance adult cardiomyocyte proliferation represents an unmet clinical need for modern cardiac regenerative medicine. Alternative approaches have been based by either analyzing in details molecular mechanisms regulating cardiomyocyte division during proficiently active developmental cardiogenesis, to much more unresponsive postnatal and adult stages, or by applying exogenous stimulation to resurge their embryonic proliferative potential. While adult mammalian cardiomyocytes show a very limited—and therapeutically irrelevant—renewal potential during adulthood (Cabrol et al., 1989; Bergmann et al., 2009), the neonatal murine heart presents remarkable regenerative activity after injury within the very first days from birth. Nevertheless, such significant renewal potential suffers a sharp decline, starting as soon as postnatal day 4 (P4) to become completely downregulated by postnatal day 7 (P7). Indeed, the 7-day-old (P7) mouse pup represents the limit of the neonatal cardiac regenerative window, being more similar to the adult heart in activating defective cardiac repair over restorative myocardial renewal (Porrello et al., 2011, 2013; Lam and Sadek, 2018). Mature cardiomyocytes seem to experience a sort of “memory loss” in their embryonic/postnatal mechanisms driving active proliferation. Therefore, defining a feasible approach to “rejuvenate” cardiomyocyte renewal program by *ad hoc* stimulation would represent a major goal in translational cardiac regenerative medicine.

In this scenario, stem- and progenitor cell–based strategies have been implemented to stimulate endogenous mechanism of cardiac regeneration *via* paracrine stimulation. Mesenchymal stromal progenitor cells obtained from leftover samples of human amniotic fluid (namely, human amniotic fluid–derived stem cells, hAFSC) are perinatal derivatives with attractive features for tissue repair. In recent years, hAFSC have been proposed as potential therapeutics given the encouraging evidence obtained by their application in several experimental disease settings (Borlongan, 2017; Loukogeorgakis and De Coppi, 2017; Bollini et al., 2018; Narakornsak et al., 2019; Fang et al., 2020). Ethical concerns associated with their isolation are minimal, since they can be easily obtained from either leftover sample from II trimester prenatal diagnosis *via* routine amniocentesis (fetal hAFSC) or from III trimester clinical waste amniotic fluid during scheduled cesarean delivery at term (perinatal hAFSC). Moreover, their remarkable high self-renewal potential and capacity to withstand long-term cryopreservation support them as appealing candidate source for regenerative medicine. Notably, hAFSC have been shown to sustain tissue recovery by exerting proliferative, anti-inflammatory, and trophic influence. The relevant paracrine effects of the fetal hAFSC secretome (namely the total amount of soluble factors that cells can secrete in their culture medium, i.e., the cell-conditioned medium, hAFSC-CM), including their released membrane-derived extracellular vesicles (hAFSC-EVs) have been assessed in preclinical models of injury, as comprehensively reviewed in Costa et al. (2022). Indeed, the emphasis on mesenchymal stromal cell–based therapy has moved toward the profiling of their secreted EVs, since they have been generally described to convey enriched trophic factors and regulating RNAs orchestrating intercellular communication and have a functional impact on target cells. This has been the case for hAFSC-EVs as well. In particular, our group has comprehensively profiled the cardioprotective potential of fetal hAFSC. We have demonstrated that fetal hAFSC secretome formulations administered in preclinical murine models of disease soon after ischemic myocardial injury (i.e., MI) or cardiotoxic insult (i.e., by means of exposure of the anthracycline drug doxorubicin), resulted in long-lasting paracrine effects preserving resident cardiomyocyte viability, while limiting fibrosis, improving local angiogenesis, rescuing cardiac function, and enhancing cardiac endogenous antioxidant defense (Lazzarini et al., 2016; Balbi et al., 2019a; Villa et al., 2021).

In this study, we wanted to investigate whether the hAFSC secretome could also exert significant cardiogenic effects, in addition to the relevant cardioprotective potential we have already observed. Since hAFSC represent “developmentally juvenile” progenitors, paracrine stimulation by their secretome may counteract postnatal loss of myocardial renewal and unlock cardiomyocyte proliferative memory. Here, we consider investigating the cardioactive influence of hAFSC secretome in the responsive neonatal murine heart model, by using a transgenic mouse with genetically driven fluorescent tracing of the different cell cycle phases based on FUCCI (fluorescent ubiquitylation–based cell cycle indicator) technology (Abe et al., 2013). While fetal hAFSC and their cardioprotective effects have been broadly characterized, a putative cardio-proliferative role of the perinatal hAFSC counterpart has not yet been defined. Hence, in order to provide comprehensive profiling of the myocardial renewal potential of the different hAFSC secretome formulations (hAFSC-CM and hAFSC-EVs), we have compared more immature II trimester fetal hAFSC, obtained from prenatal screening, over III trimester perinatal hAFSC obtained at term, in order to identify the most suitable perinatal derivative for future translational studies.

## 2 Materials and Methods

### 2.1 R26pFUCCI2 Mouse Model

R26pFUCCI2 mice [(CDB0203T) http:www.clst.riken.jp/arg/reporter_mice.html], first described in Abe et al. (2013), were obtained following MTA (material transfer agreement) with Dr. Yasuhide Furuta, RIKEN Center for Life Science Technologies and provided as courtesy of Prof. Shrinivas Shankar and Prof. Paul Riley from University of Oxford, United Kingdom. FUCCI (fluorescent ubiquitylation–based cell cycle indicator) technology allows the discrimination of cell cycle phases (G1 versus S/G2/M) by dual color imaging *via* a nuclear fluorescent signal. R26pFUCCI mice constitutively express *mCherry-hCdt1* and *mVenus-hGeminin* constructs as a single transgene under the Rosa26 promoter. Cdt1, chromatin licensing and DNA replication factor 1, is a key regulator in the assembly of pre-DNA replication complexes in the G1 phase, and it is controlled during the S phase by the protein Geminin, which inhibits it, thus guaranteeing that DNA is replicated only once in each cell cycle (Wohlschlegel et al., 2000). Therefore, this transgenic model licenses the distinction between cells in the G1 phase, as labeled with mCherry fluorescent red nuclear expression, from those transitioning through the S phase into mitosis by the mVenus green nuclear signal (Abe et al., 2013). Mice were housed and maintained in a controlled environment in the Animal Facility at IRCCS Ospedale Policlinico San Martino, Genova, Italy, according to International Standards of Animal Welfare, as ruled by the Italian Ministry of Health (DM.146/2009-A). Animal maintenance, handling, and all procedures were performed in strict compliance to all applicable international, national, and/or institutional guidelines for the care and use of animals (D. Lgs. 26/2014, D. Lgs. 116/92, legislative transposition of Directive 2010/63/EU of European Parliament and of Council of 22 September 2010). Heterozygous R26pFUCCI2^+/−^ mice were obtained from crossing R26pFUCCI2^+/−^ males with wild-type C57Bl6/J or FVB females, according to specific animal license authorization (62/2019-PR and 230/2019-PR from the Italian Ministry of Health). Mice genotype was assessed using the Phire Tissue Direct PCR Master Mix (Thermo Fisher Scientific), following manufacturer’s instructions. Primer sequences are available on request.

### 2.2 *In vitro* Cell Culture

#### 2.2.1 Human Amniotic Fluid-Stem Cell Isolation and *in vitro* Culture

Human amniotic fluid stem cells (hAFSC) were isolated from leftover samples of human amniotic fluid (hAF) collected during routine amniocentesis at II trimester gestation for prenatal screening (fetal hAFSC), or as clinical waste material (perinatal hAFSC) from scheduled cesarean section delivery in the III trimester, from the Prenatal Diagnosis Perinatal Medicine Unit, IRCCS Ospedale Policlinico San Martino, from the Fetal and Perinatal Medical and Surgery Unit, and from the Human Genetics Laboratory, IRCCS Istituto Giannina Gaslini Hospital, in Genova, Italy. All donors provided informed written consent for research use of the leftover hAF specimen, according to authorization previously granted from the local ethical committee (P.R. 428REG 2015) and in compliance with Helsinki Declaration guidelines. II trimester fetal hAF samples were obtained from female donors with average age of 38.30 ± 0.40 years old (*n* = 32); III trimester perinatal hAF samples were obtained from female donors with average age of 34.25 ± 1.31 years old (*n* = 10). Clusters of adherent mesenchymal stromal cells (MSC) were obtained from hAF samples (hAF-MSC). hAF-MSC expressing normal karyotype were further processed by immunomagnetic sorting for c-KIT expression (CD117 MicroBead Kit, Miltenyi Biotechnology) to obtain c-KIT^+^ hAFSC, as previously reported (Lazzarini et al., 2016; Balbi et al., 2017, 2019b; Costa et al., 2021; Villa et al., 2021). c-KIT^+^ hAFSC (here indicated as hAFSC) were cultured in the minimal essential medium (MEM)-alpha with 15% fetal bovine serum (FBS, Gibco—Thermo Fisher Scientific), 18% Chang B and 2% Chang C medium (Irvine Scientific), 1% l-glutamine, and 1% penicillin/streptomycin (Gibco—Thermo Fisher Scientific) in incubator at 37°C with 5% CO_2_ and 20% O_2_ atmosphere. hAFSC were cultured up to five passages *in vitro* before being primed to isolate their secretome.

#### 2.2.2 Mouse Neonatal Ventricular Cardiomyocyte Isolation and Culture

Primary cultures of R26pFUCCI2 neonatal mouse ventricular cardiomyocytes (mNVCM) were obtained from 1-day-old murine heart tissue. Neonatal mice were obtained by crossing R26pFUCCI2^+/−^ males with wild-type FVB females (according to authorization n. 62/2019-PR). mNVCM were isolated by enzymatic digestion with papain and thermolysin enzymes using the Pierce Primary Cardiomyocyte Isolation Kit (Thermo Fisher Scientific), following manufacturer’s instructions. Primary R26pFUCCI2^+/−^ mNVCM were seeded at density of 0.25 × 10^6^ cells/cm^2^ on either an 8-well-PCA (PolyCycloAlkanes) detachable chamber slide (Sarsted, Germany) or on a 24-well plate coated with a 0.2% gelatin (Sigma-Aldrich) and fibronectin (1:1000 dilution) solution. mNVCM were cultured in DMEM for primary cell (Thermo Fisher Scientific) with 10% heat-inactivated fetal bovine serum, (FBS, Gibco—Thermo Fisher Scientific) and 1% penicillin/streptomycin (Gibco—Thermo Fisher Scientific) in incubator at 37°C with 5% CO_2_ and 20% O_2_ atmosphere.

### 2.3 Isolation and Concentration of hAFSC Secretome Formulations

Fetal and perinatal hAFSC were seeded at 2,000 cells/cm^2^ in T-75 and T-150 tissue-culture flasks (Eppendorf). Cells were cultured *in vitro* under normoxic (20% O_2_, 5% CO_2_ at 37°C) or hypoxic (1% O_2_, 5% CO_2_ at 37°C) conditions in CellXpert^®^ C170i and Galaxy^®^ 48R CO_2_ Eppendorf incubators for 24 h in a serum-free medium (SF: high glucose DMEM, supplemented with 1% l-glutamine and 1% penicillin/streptomycin, Gibco—Thermo Fisher Scientific), as a priming strategy to enhance the secretion of cardioactive paracrine factors, as we previously reported (Lazzarini et al., 2016; Balbi et al., 2017, 2019b; Costa et al., 2021; Villa et al., 2021). Fetal and perinatal hAFSC–conditioned medium (fetal and perinatal hAFSC-CM) was collected, centrifuged at 300 × g for 10 min and 2,000 × g for 20 min to remove cell debris, and further concentrated by means of ultrafiltration using 3 kDa selective cutoff membranes (Amicon Ultra-15, Millipore) at 3,000 × g for 90 min.

hAFSC-EVs were separated and concentrated by serial ultracentrifugation from hAFSC-CM. In brief, hAFSC-CM was centrifuged at 10,000 × g for 40 min; pellet was then discarded, and the liquid phase was further processed by ultracentrifugation in an Optima L-90K at 100,000 × g for 120 min using a swinging-bucket SW55Ti rotor (all Beckmann Coulter). The pellet containing the separated and concentrated hAFSC-EVs was then washed in phosphate-buffered saline (1X PBS) solution by a final step of centrifugation at 100,000 × g for 120 min and then resuspended in 0.22 µm filtered 1X PBS. Concentration of proteins within hAFSC-CM and on hAFSC-EV surface was assessed using the bicinchoninic acid (BCA) assay (Thermo Fisher Scientific); the albumin (BSA) solution provided by the manufacturer was diluted in PBS to prepare protein standards with defined concentration (5, 25, 50, 125, and 250 μg/ml) to set the reference calibration curve for a specific working range hAFSC secretome formulations, and the control SF vehicle medium were diluted in PBS and incubated with the BCA working reagent solution, according to the manufacturer’s instruction. Samples were acquired on a Gen5 Microplate Reader (Agilent Technologies) at wavelength 570 nm to evaluate hAFSC-CM and hAFSC-EV yield. A nanoparticle tracking analysis (NTA) on hAFSC secretome formulations was performed on the ZetaView analyzer (Particle Metrix) in order to assess EV particle amount in the secretome dosage used for the *in vitro* experiments.

### 2.4 *In vitro* Stimulation of mNVCM by Fetal Versus Perinatal hAFSC Secretome Formulations

Isolated R26pFUCCI2 mNVCM were seeded as 0.25 × 10^6^ cells/cm^2^ on 8-well-PCA detachable chamber slides (Sarsted, Germany), corresponding to 200,000 mNVCM in 200 μl of media per well. Furthermore, 48 h after seeding, cells were stimulated by 80 μg/ml fetal or perinatal hAFSC-CM or by 5 μg/ml fetal or perinatal hAFSC-EVs for further 48 h in SF conditions (DMEM for primary cell with 1% penicillin/streptomycin, all Gibco—Thermo Fisher Scientific). Vehicle solution (SF medium) was considered as control condition (Ctrl) treatment. The hAFSC secretome dosage employed for these experiments was based on our previous findings about their stimulatory potential (Balbi et al., 2019a; 2019b) further details are available on request. In each well, mNVCM were treated with 16 μg hAFSC-CM or 1 μg hAFSC-EVs in 200 μl SF final medium volume. The hAFSC-CM and hAFSC-EV dosages were enriched with a comparable amount of EV particles (hAFSC-CM: 5.82 × 10^8^ ± 4.43 × 10^8^ particles; hAFSC-EVs: 8.19 × 10^8^ ± 1.60 × 10^8^ particles, p > 0.05, Supplementary Figure S1A). Schematic of *in vitro* experimental design is reported in Supplementary Figure S1B. Cardiomyocyte cell cycle progression was evaluated 72 h after hAFSC secretome stimulation by immunostaining. A FUCCI nuclear signal was correlated with expression of cardiomyocyte-specific sarcomeric markers (cardiac alpha-actinin and cardiac troponin I); mNVCM were fixed in 4% paraformaldehyde (PFA) solution (Sigma Aldrich) and then incubated with a primary antibody against GFP (green fluorescent protein, ab13970, Abcam) followed by Alexa Fluor 488–conjugated secondary antibody (A-11039, Thermo Fisher Scientific) to enhance the mVenus probe signal, indicating progression through S up to M phases, and with an anti-sarcomeric cardiac alpha-actinin primary antibody (αAct, ab9465, Abcam) followed by Alexa Fluor 594–conjugated secondary antibody (A-11032, Thermo Fisher Scientific). To detect cytokinetic events, a primary antibody against Aurora B kinase, a chromosomal passenger protein binding to protein involved in cleavage furrow and midbody formation, (AuBK, ab2254, Abcam) followed by Alexa Fluor 647–conjugated secondary antibody (A-32733, Thermo Fisher Scientific) was used. The paracrine potential of fetal versus perinatal hAFSC-CM and hAFSC-EVs to support cardiomyocyte cell cycle progression was assessed as the percentage of αAct-positive cells (mNVCM) expressing nuclear mVenus signal (as enhanced by anti-GFP antibody) over the total αAct-positive cells by counting at least five independent fields in each condition at ×20 magnification. Cytokinesis events were detected as percentage of αAct-positive cells (mNVCM) expressing Aurora B kinase localizing at the mid-body by counting at least five independent fields in each condition at ×40 magnification. In a second set of experiments, R26pFUCCI2^+/−^ mNVCM were treated with a YAP pathway inhibitor [10 μM Verteporfin solution, Tocris, as from (Bassat et al., 2017)] prior to hAFSC secretome stimulation. R26pFUCCI2 mNVCM were treated with or without YAP inhibitor in the SF medium for 48 h and treated with 5 μg/ml hAFSC-EVs (corresponding to 1 μg hAFSC-EVs for 200,000 mNVCM in 200 μl of final medium volume) for further 48 h (as illustrated in schematic in Supplementary Figure S1C) in the SF medium. mNVCM were analyzed at 72 h posttreatment. mNVCM were fixed in 4% paraformaldehyde solution (Sigma Aldrich) and incubated with primary antibody against GFP (ab13970, Abcam) followed by Alexa Fluor488–conjugated secondary antibody (A-11039, Thermo Fisher Scientific) and with anti-cardiac troponin I primary antibody (TnI, ab47003, Abcam) followed by Alexa Fluor 647–conjugated secondary antibody (A-32733, Thermo Fisher). Immunostaining images were acquired on an Axiovert microscope equipped with Axiovision software (Carl Zeiss). Signal intensity was analyzed and measured with ImageJ software [https://imagej.nih.gov/ij/, (ImageJ, 2021)]. Cardiomyocyte cell cycle progression was assessed as the percentage of cTnI-positive cells (mNVCM) expressing a nuclear mVenus signal (as enhanced *via* anti-GFP antibody) over the total cTnI-positive cells by counting at least five independent fields in each condition at ×20 magnification.

### 2.5 Gene Expression Profile of mNVCM

The gene expression profile of R26pFUCCI2^+/−^ mNVCM stimulated by hAFSC secretome over vehicle solution-treated cells as control was investigated using real-time qRT-PCR. mNVCM were seeded on 24-well plate as 500,000 cells/well and then stimulated by 5 μg/ml hAFS-EVs (corresponding to 2 μg per well in 400 μl of final medium volume). Total RNA from mNVCM stimulated with hAFSC secretome over vehicle-treated ones was extracted using the Qiazol Lysis Reagent (Qiagen), according to manufacturer’s indications. cDNA was obtained by using the iScript Reverse Transcription Supermix for RT-qPCR (Bio-Rad). Real-time qRT-PCR was carried out on a 7500 Fast Real-Time PCR System (Applied Biosystems) using the Syber Green Master Mix (BrightGreen 2X qPCR, Abm). The following primer sequences were used: mouse *cofilin-2* (*Cfl2*, forward: 5′-CCG​ACC​CCT​CCT​TCT​TCT CG-3′ and reverse: 5′-GTA​ACT​CCA​GAT​GCC​ATA​GTG C-3′) and mouse *beta-2 microglobulin* as a housekeeping reference (*β2M*, forward: 5′-CTG​CTA​CGT​AAC​ACA​GTT​CCA CCC-3′ and reverse: 5′-CAT​GAT​GCT​TGA​TCA​CAT​GTC​TCG-3′). *Cfl2* expression was evaluated by means of the 2^−ddCt^ method with vehicle solution–treated control mNVCM (Ctrl) as a calibrator.

### 2.6 Evaluation of F-Actin and G-Actin in mNVCM

To investigate mNVCM cytoskeleton re-arrangement following stimulation with the hAFSC secretome over vehicle solution as control, the amount of polymerized F-Actin over monomeric G-Actin was analyzed as indication of cardiomyocyte cell cycle progression (Torrini et al., 2019). mNVCM were seeded on a 24-well plate as 500.000 cells/well, which were then stimulated by 5 μg/ml hAFSC-EVs (corresponding to 2 μg per well in 400 μl of medium final volume). The F-Actin/G-Actin *In Vivo* Assay Biochem Kit (Cytoskeleton) was used as per manufacturer’s instructions. mNVCM proteins were boiled with Laemmli SDS sample buffer 6x (VWR International), separated on 4–20% Mini-PROTEAN®TGX™ Precast Gel, and transferred onto a polyvinylidene difluoride (PVDF) membrane with a semi-dry transfer system (all from Bio-Rad). Membrane were incubated with F-Actin/G-Actin antibody provided in the assay and then with IRDye^®^ 800CW goat anti-rabbit secondary antibody (LI-COR Biosciences). Infrared signals were detected using the Odyssey CLx Detection System (LI-COR Biosciences). Quantification was performed using an Odyssey CLx analyzer (LI-COR Biosciences).

### 2.7 Analysis of hAFSC Secretome Cardioactive Potential in a Preclinical Neonatal Mouse Model of Myocardial Infarction

Transgenic R26pFUCCI2 neonatal mice were obtained by crossing heterozygous R26pFUCCI^+/−^ males with C57Bl6/J wild-type females. 4-day-old (P4) and 7-day-old (P7) R26pFUCCI2 pups were used for *in vivo* experiments in strict compliance with national and European international standards of animal care and according to required authorization from the Italian Ministry of Health (authorization n. 230/2019-PR). The preclinical mouse model of myocardial infarction (MI) was performed according to Del Campo et al. (2022). In brief, neonatal mice were anesthetized under analgesia by an initial step of inhalation of 3% isoflurane in oxygen, followed by exposure to a short period of controlled hypothermia. MI was performed by permanent ligation of the left anterior descending coronary artery (LAD). In the sham control group (Sham MI) animals underwent the same surgical procedure without coronary artery ligation. In treated mice, soon after coronary ligation, 4.5 μg fetal hAFSC-EVs in 20 μl of 1X PBS solution (fetal hAFSC-EVs) versus an equal volume of vehicle solution (1X PBS, Ctrl) were administered by intraperitoneal injections. The indicated hAFSC-EV dosage was based on our previous findings (Balbi et al., 2019a). Hearts were harvested after 3 and 7 days from MI, and the three experimental groups were assessed by histological and immunostaining analyses (Sham MI: *n* = 4 for each stage and time point analysis; fetal hAFSC-EVs: *n* = 8 for each stage and time point analysis; Ctrl: *n* = 8 for P4 pups at day 3 and day 7; fetal hAFSC-EVs: *n* = 8 for P7 at day3; Ctrl: *n* = 9 for P7 pups at day 3; *n* = 8 for P7 pups at day 3 and day 7). Cardiac tissue was fixed in 4% paraformaldehyde (PFA) solution, incubated in a 30% sucrose–PBS solution overnight, and snapped-frozen in liquid nitrogen for cryo-sectioning. Furthermore, 6-μm cryo-sections were processed for hematoxylin and eosin staining to confirm myocardial injury. Immunostaining was performed to evaluate resident cardiomyocytes re-entering the cell cycle by using the primary antibody against cardiac troponin I (cTnI, ab47003, Abcam) followed by Alexa Fluor 647–conjugated secondary antibody (A-32733, Thermo Fisher Scientific), together with a primary antibody against green fluorescent protein (GFP, ab13970, Abcam) followed by Alexa Fluor 488–conjugated secondary antibody (A-11039, Thermo Fisher Scientific) to enhance the mVenus nuclear signal. Immunostaining images were acquired on an Axiovert microscope equipped with Axiovision software (Carl Zeiss). Signal intensity was analyzed and measured with ImageJ software [https://imagej.nih.gov/ij/, (ImageJ, 2021)]. mVenus-positive and cTnI-positive resident cells (S-M phase cardiomyocytes) were evaluated as cells/field by counting at least five independent fields for each tissue section at ×20 magnification.

### 2.8 Characterization of hAFSC-EVs

#### 2.8.1 Bioinformatic Analysis of miRNA Profile

Bioinformatic analysis of miRNA predicted targets was performed on RNA sequencing data obtained from the comprehensive fetal hAFSC-EV profiling previously performed (Costa et al., 2021); results of multiple sources were aggregated using the miRNAtap R package (Package “miRNAtap”, 2022 Title miRNAtap: microRNA Targets-Aggregated Predictions, 2022).

#### 2.8.2 Western Blotting

Total protein from hAFSC-EVs was extracted by lysing samples with ice-cold RIPA (radioimmunoprecipitation assay) buffer supplemented with SIGMA FAST™ Protease Inhibitors and Phosphatase Inhibitor Cocktail 3 and 2 (all from Sigma). Total protein concentration was determined using the QuantiPro™ BCA Assay Kit (Sigma). Proteins were boiled with Laemmli SDS sample buffer 6x (VWR International), separated on 4–20% Mini-PROTEAN®TGX™ Precast Gel, and transferred onto a PVDF membrane with a semi-dry transfer system (all from Bio-Rad Europe).

hAFSC-EVs were characterized for the expression of canonical markers of small EVs. Membranes were incubated with rabbit anti-TSG101 (Tumor Susceptibility Gene 101) antibody (ab125011, Abcam); rabbit anti-Syntenin-1 antibody (ab19903, Abcam) and rabbit anti-ALIX (ALG-2-interacting protein X) antibody (ab186429, Abcam). A rabbit anti-GRP94 (Glucose-Regulated Protein 94) antibody (ab238126, Abcam) was used as contamination control. Agrin (Agrn) protein expression was investigated in fetal and perinatal hAFSC and in their corresponding secreted EVs. As normalizing control, Syntenin-1 was considered as small EV reference marker (Kugeratski et al., 2021). Membranes were incubated with primary rabbit anti-Agrin antibody (ab85174, Abcam) and primary rabbit anti-Syntenin-1 antibody (ab133267, Abcam). Membranes were following incubated with IRDye^®^ 800CW goat anti-rabbit secondary antibody (LI-COR Biosciences, Lincoln, Nebraska, United States). Infrared signal was detected using Odyssey CLx Detection System (LI-COR Biosciences). Quantification was performed using Odyssey CLx analyzer (LI-COR Biosciences).

#### 2.8.3 Flow Cytometry Analysis

The presence of CD9, CD81 and CD63 tetraspanin antigens on hAFSC-EV surface was also evaluated by flow cytometry, according to the protocol optimized by (Gorgun et al., 2019). Fetal and perinatal hAFSC-EVs were labeled with the lipophilic fluorescent dye CFSE (CFSE: Vybrant CFDA SE Cell Tracer Kit, Invitrogen) according to the manufacturer’s instructions in combination with one of the following fluorochrome-conjugated antibodies for 15 min at room temperature: PE-Cy7™-conjugated mouse anti-human CD63; PE-conjugated mouse anti-human CD9; BV421-conjugated mouse anti-human CD81; PE-Cy7™-conjugated mouse IgG1 κ and PE-mouse IgG1, Clone 40 isotype controls (all BD Biosciences). Fetal and perinatal hAFSC-EVs (*n* = 3) were analyzed on a BD FACSAria II equipped with three lasers (405, 488 and 640 nm) and data analyzed by FACSDiva software, v8.0 (all BD Biosciences). Instrument setting was defined by using a mixture of fluorescent beads of varying diameters [Megamix-Plus FSC and Megamix-Plus SSC (BioCytex)] following manufacturer’s instructions.

### 2.9 Statistical Analyses

All values are presented as mean ± s.e.m. (standard error of mean) of at least three (*n* = 3) independent experiments. Comparisons were drawn by one-way ANOVA followed by *post hoc* Tukey’s multiple comparisons test for mNVCM stimulation or by *t*-Test when comparing fetal versus perinatal hAFSC-CM/EVs. Statistical analysis was performed using GraphPad Prism Version 8.0.2 (GraphPad Software) with statistical significance set at *p < 0.05.

## 3 Results

### 3.1 Fetal hAFSC-EVs Sustain mNVCM Cell Cycle Re-Entry and Renewal *in vitro*

**FIGURE 1:**
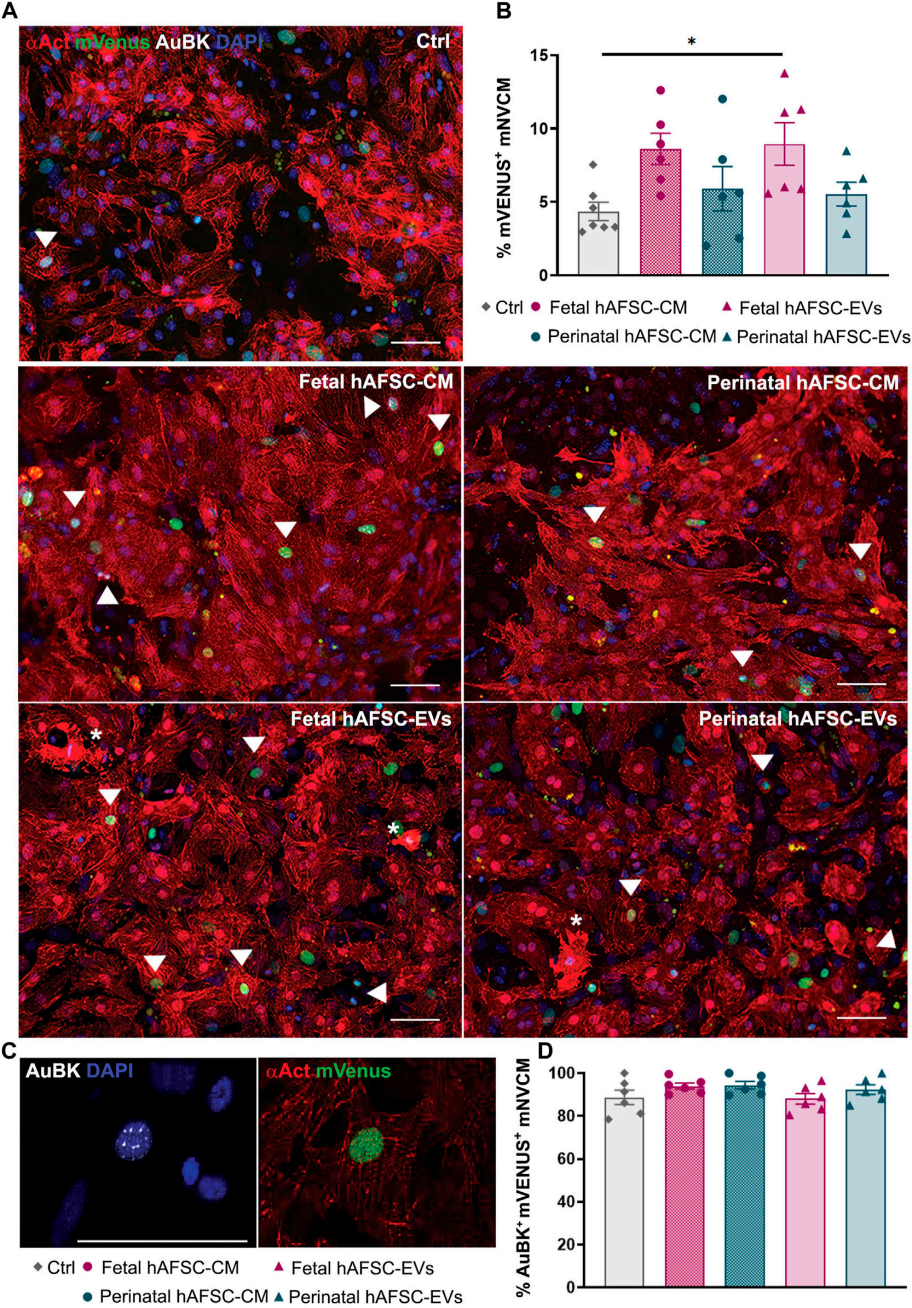
*In vitro* mNVCM cell cycle re-entry following stimulation by fetal versus perinatal hAFSC secretome formulations. **(A)** Representative images of R26pFUCCI2^+/−^ mNVCM in vehicle-treated control conditions (SF medium, Ctrl) or following treatment with fetal or perinatal hAFSC-CM or hAFSC-EVs by immunostaining for sarcomeric alpha-actinin (αAct, red), mVenus-Geminin (mVenus, green), Aurora B kinase (AuBK, white), and DAPI (blue), scale bar: 50 µm. White arrows point at αAct-positive cells (mNVCM) with mVenus nuclear signal; white asterisks indicate mNVCM with disarranged α-actinin expression as sarcomeric disassembly feature. **(B)** Analysis of mVenus-positive and αAct-positive cells (mVenus^+^ mNVCM) expressing αAct cells following stimulation with fetal or perinatal hAFSC secretome formulations over vehicle-treated cells (Ctrl). All values are expressed as mean ± s.e.m percentage mVenus-positive and αAct-positive cells (% mVenus^+^ mNVCM) of independent experiments (Ctrl: 4.36 ± 0.62%, *n* = 7; fetal hAFSC-CM: 8.62 ± 1.06%, *n* = 6; perinatal hAFSC-CM: 5.90 ± 1.51%, *n* = 6; fetal hAFSC-EVs: 8.95 ± 1.45%, *n* = 6; perinatal hAFSC-EVs: 5.54 ± 0.80%, *n* = 6; *p = 0.0455). **(C)** Representative image of nuclear AuBK expression co-localizing with mVenus signal in αAct-positive cells (mNVCM); scale bar: 50 µm. **(D)** Evaluation of mVenus-positive and αAct-positive cells showing AuBK nuclear signal (AuBK^+^ mVenus^+^ mNVCM) following stimulation with fetal versus perinatal hAFSC secretome formulations over vehicle-treated control condition (Ctrl). All values are expressed as mean ± s.e.m. percentage of AuBK-positive and mVenus-positive mNVCM (%AuBK^+^ mVenus^+^ mNVCM) of *n* = 6 independent experiments (Ctrl: 88.73 ± 3.39%; fetal hAFSC-CM: 93.99 ± 1.44%; perinatal hAFSC-CM: 94.37 ± 1.76%; fetal hAFSC-EVs: 88.06 ± 2.50%; perinatal hAFSC-EVs: 92.26 ± 2.23%).

The myocardial renewal potential of the fetal versus perinatal hAFSC-CM and hAFSC-EV formulations was first evaluated *in vitro* by stimulating R26pFUCCI2^+/−^ mNVCM with 80 μg/ml of hAFSC-CM and 5 μg/ml of hAFSC-EVs over vehicle control solution (Ctrl, SF medium). Primary culture of R26pFUCCI2^+/−^ mNVCM showed 70% enrichment of sarcomeric alpha-actinin (αAct)-positive cardiomyocytes over contaminating cardiac cells (i.e., cardiac fibroblasts, epicardial and endothelial cells as obtained from enzymatic digestion of the neonatal heart). mNVCM were evaluated by immunostaining as the percentage of αAct-positive cells with mVenus probe nuclear signal as engaging of cell cycle activity from phase S up to M, (Figure 1A and corresponding Supplementary Figure S2). While stimulation by the fetal hAFSC-CM resulted in a positive trend in the proportion of mNVCM re-entering the cell cycle, fetal hAFSC-EVs induced a significant 2.0-fold increase (*p < 0.05) in the progression into M phase, when compared to untreated cells (Ctrl). On the contrary, the secretome formulations obtained from the more mature perinatal hAFSC did not exert relevant cardiogenic effect.

**FIGURE 2:**
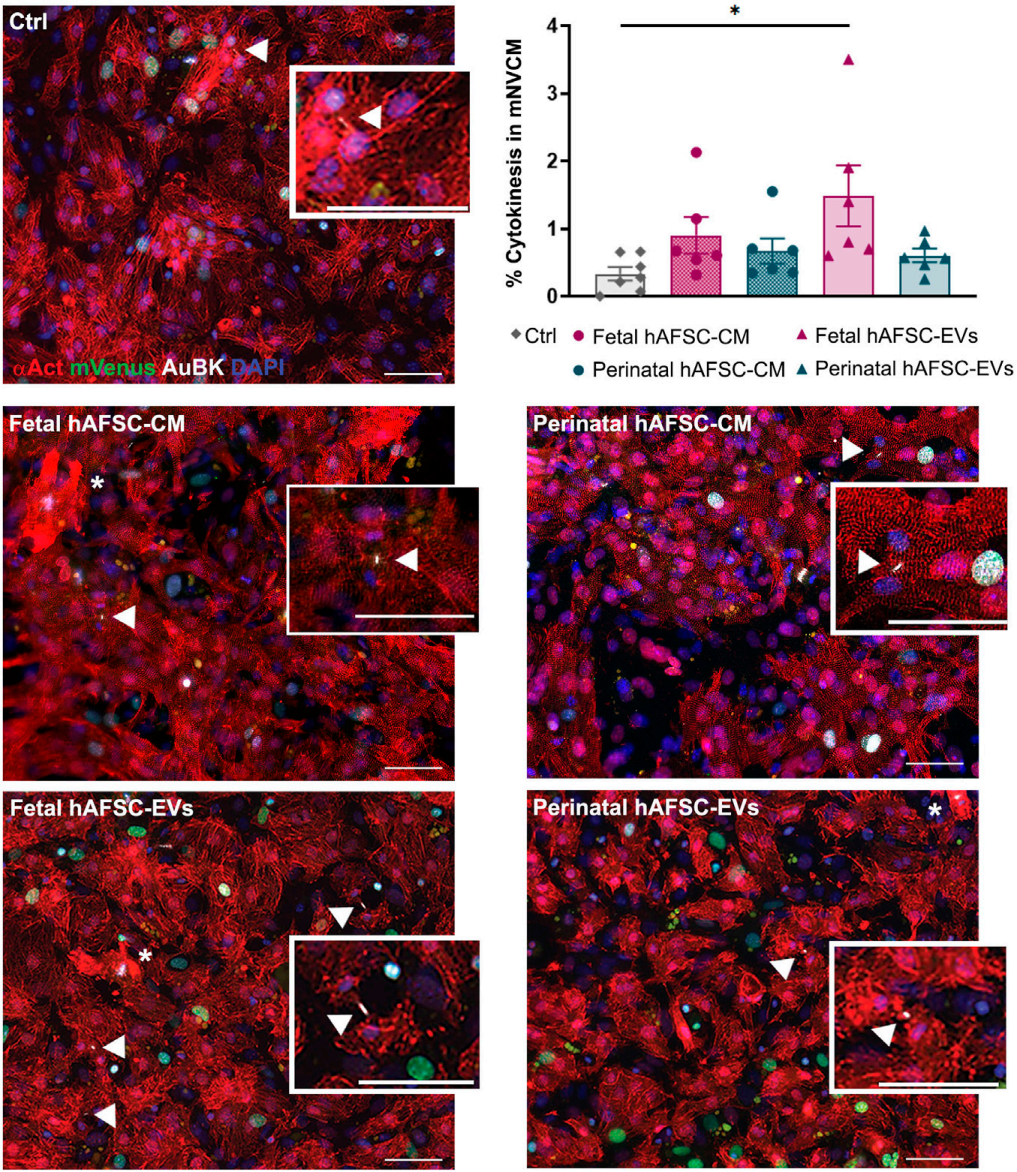
*In vitro* cytokinesis of mNVCM following stimulation by fetal versus perinatal hAFSC secretome formulations. Representative images of AuBK localization at cell mid-body in R26pFUCCI2^+/−^ mNVCM in vehicle-treated control conditions (SF medium, Ctrl) or following treatment with fetal or perinatal hAFSC-CM or hAFSC-EVs by immunostaining for sarcomeric α-actinin (αAct, red), mVenus-Geminin (mVenus, green), Aurora B kinase (AuBK, white), and DAPI (blue), scale bar: 50 µm. White arrows indicate AuBK expression at cell mid-body, corresponding to the completion of cytokinesis in αAct-positive cells (mNVCM), as illustrated in the inlet magnification, scale bar: 50 µm; white asterisks indicate mNVCM with disarranged α-actinin expression as sarcomeric disassembly feature. Upper right panel: evaluation of αAct-positive cells (mNVCM) with AuBK expression at cleavage furrows following stimulation with fetal versus perinatal hAFSC secretome formulations over vehicle-treated control condition (Ctrl). All values are expressed as mean ± s.e.m. percentage of dividing cardiomyocytes (% Cytokinesis in mNVCM) in independent experiments (Ctrl: 0.33 ± 0.09%, *n* = 7; fetal hAFSC-CM: 0.90 ± 0.27%, *n* = 6; perinatal hAFSC-CM: 0.67 ± 0.18%, *n* = 6; fetal hAFSC-EVs: 1.48 ± 0.45%, *n* = 6; perinatal hAFSC-EVs: 0.60 ± 0.10%, *n* = 6; *p = 0.0202).

We then evaluated the expression of Aurora B kinase (AuBK) enzyme, a mitotic checkpoint for appropriate chromosome segregation during cell proliferation. We noticed that regardless the treatment received, the great majority (i.e., from 88% up to 94%) of mNCVM with mVenus nuclear also co-expressed AuBK within the nucleus (Figure 1B). Localization of AuBK in well-developed cleavage furrow and at middle stage midbody in between mNVCM was then analyzed as indication of commitment to cytokinesis and cell division; a 4.5-fold increase in the percentage of mNVCM (αAct-positive cells) expressing AuBK at midbody was detected only when cells were stimulated by fetal hAFSC-EVs (*p < 0.05, Figure 2 and corresponding Supplementary Figure S3), over vehicle control treatment.

### 3.2 Fetal hAFSC-EVs Influence Cytoskeleton Re-Organization in mNVCM

**FIGURE 3:**
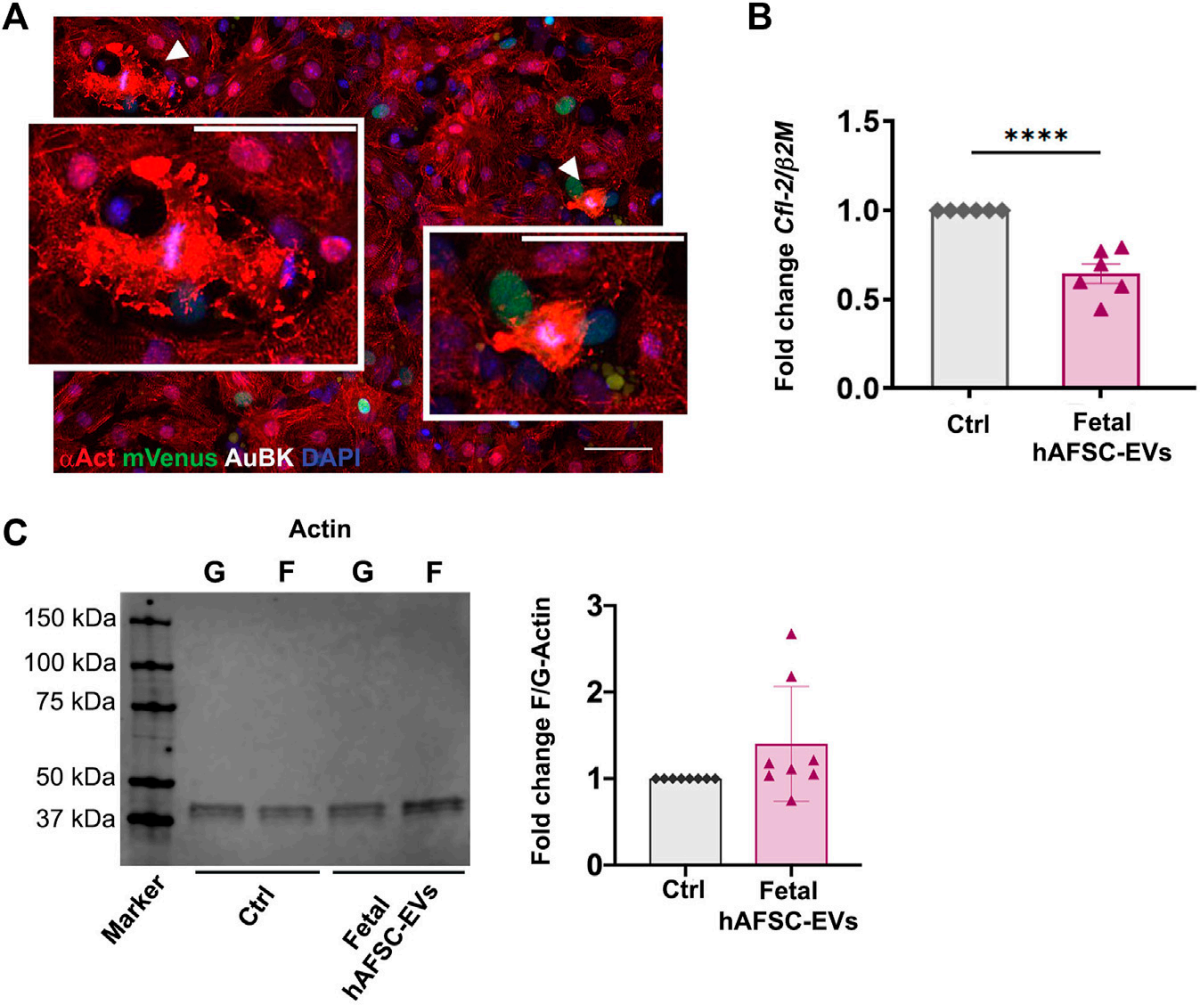
Analysis of actin cytoskeleton regulation in mNVCM following fetal hAFSC-EV stimulation **(A)** Representative immunostaining pictures of mNVCM with sign of sarcomeric disassembly following fetal hAFSC-EV treatment as seen in Figure 1A; white arrows indicate mNVCM with disarranged expression of sarcomeric α-actinin (αAct, red) and intense Aurora B kinase signal (AuBK, white) associated to the chromosomal material (DAPI, blue), scale bar: 50 and 25 µm in the inlet. **(B)** Real-time qRT-PCR analysis of mNVCM stimulated by fetal hAFSC-EVs over vehicle-treated control cells (Ctrl) for *Cofilin-2 (Cfl-2)* gene expression as normalized to Beta 2 Microglobulin (β2M), as housekeeping reference. All values are expressed as mean ± s.e.m of *CFL-2/β2M* fold change for *n* = 6 independent experiments with Ctrl group as calibrator (Ctrl: 1; fetal hAFSC-EVs: 0.64 ± 0.13; ****p < 0.001). **(C)** Left panel: representative image of Western Blot analysis of G-Actin (G) and F-Actin (F) expression in mNVCM treated with fetal hAFSC-EVs (fetal hAFSC-EVs) over vehicle solution control (Ctrl); right panel: evaluation of F-Actin/G-Actin in mNVCM treated with fetal hAFSC-EVs over vehicle solution control. All values are expressed as mean ± s.e.m of fold change of F-Actin/G-Actin ratio (Fold Change F/G-Actin) of *n* = 8 independent experiments by densitometry on protein band intensity with Ctrl group as calibrator (Ctrl: 1; fetal hAFSC-EVs: 1.40 ± 0.23). kDa: kilo Dalton*.*

Some mNVCM treated with hAFSC secretome formulations showed disorganized expression of cardiac alpha-actinin (Figure 1A and Figure 2, with details at higher magnification in Figure 3A, and corresponding Supplementary Figures S2, S3), suggesting cardiomyocyte sarcomeric disassembly, as required for cell cycle re-entry. In these mNVCM, AuBK localized within the nucleus overlapping the DAPI signal as suggesting being associated with chromosomal material at mitotic metaphase/anaphase (Figure 3A). Since immunostaining analyses defined fetal hAFSC-EVs as the most influencing secretome formulation, we further investigated their paracrine effect on mNVCM cytoskeleton re-organization. mNVCM treated with fetal hAFSC-EVs showed a significant decrease with a 0.6-fold (****p < 0.0001) gene expression of *Cofilin-2* (*Cfl-2*), a modulator of actin cytoskeleton antagonizing cardiomyocyte proliferation (Torrini et al., 2019), over vehicle-treated Ctrl cells considered as reference (Figure 3B). Fetal hAFSC-EVs are enriched with microRNAs (miRNAs) within their cargo, as we have recently reported in (Costa et al., 2021). Additional bioinformatic analysis, suggested that some of the mostly enriched miRNAs within fetal hAFSC-EVs (miR-93-5p; miR-152-3p; miR-200b-3p; miR-429; miR-199a-3p; miR-20a-5p; miR-425-5p) preferentially target the *CFL-2* gene (Supplementary Figure S4A). Cofilin-2 regulates the actin cytoskeleton by promoting conversion of polymerized F-Actin back into monomeric G-Actin (Torrini et al., 2019); we then assessed the ratio between G-Actin and F-Actin in treated mNVCM; cells stimulated by fetal hAFSC-EVs showed a mild trend in the increase of polymerized F-Actin compared to the monomeric G form, as evaluated by the F/G ratio fold change (Figure 3C).

### 3.3 Fetal hAFSC-EVs can Sustain Resident Cardiomyocyte Cell Cycle Progression in a Preclinical Neonatal Mouse Model of Myocardial Infarction in the Short Term

The cardioactive paracrine potential of fetal hAFSC-EVs was evaluated in a 4-day-old (P4) and 7-day-old (P7) R26FUCCI2^+/−^ neonatal mouse model of myocardial infarction (MI), at 3 and 7 days post injury. The myocardium of R26pFUCCI2^+/−^ pups was evaluated by immunostaining for the expression of cardiac troponin I (cTnI)-positive cardiomyocytes with nuclear signal by the mVenus probe marking the engaging of cell cycle activity (from phase S up to completion of phase M).

**FIGURE 4:**
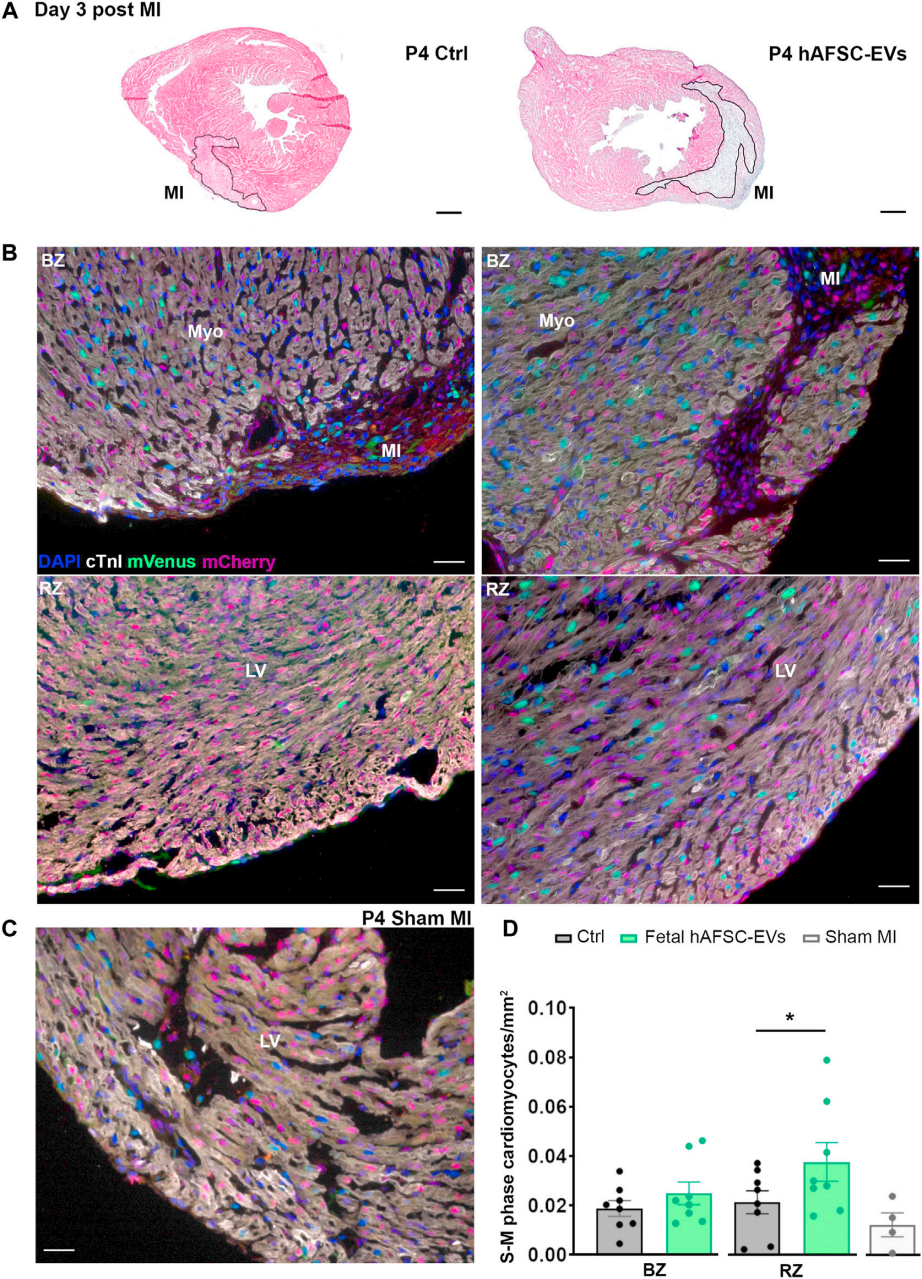
In vivo cardiogenic effect of fetal hAFSC-EVs on P4 neonatal mouse heart at 3 days post MI. **(A)** Representative pictures of hematoxylin and eosin staining on cryo-sections of R26pFUCCI2^+/−^ 4-day-old (P4) mouse myocardial tissue in: vehicle-treated control condition (PBS solution, left panel, Ctrl) and following intraperitoneal injection of 4.5 μg fetal hAFSC-EVs (hAFSC-EVs, right panel); myocardial infarct area (MI) is traced in black, scale bar: 300 µm. **(B)** Representative pictures of immunostaining analysis on cryo-sections of R26pFUCCI2^+/−^ 4-day-old (P4) mouse myocardial tissue in: vehicle-treated control condition (PBS solution, left panel, Ctrl), following intraperitoneal injection with 4.5 μg fetal hAFSC-EVs (hAFSC-EVs, right panel), or in Sham control condition [**(C)**, Sham MI] for DAPI (blue), cardiac troponin I (cTnI, white), mVenus-Geminin (mVenus, green), and mCherry-Cdt1 (mCherry, red), at 3 days post MI (P4 d3); scale bar: 50 µm. **(D)** Evaluation of mVenus-positive and cTnI-positive resident cells (S-M phase cardiomyocytes/mm^2^) within the myocardial tissue of mice receiving fetal hAFSC-EVs treatment compared to vehicle Ctrl solution in the MI border zone (BZ, upper panel) and in the remote zone (RZ, lower panel) of the left ventricle (LV). All values are expressed as mean ± s.e.m. of cTnI-positive cardiomyocytes expressing mVenus-positive nuclei per mm^2^ (P4 d3 Ctrl BZ: 0.01879 ± 0.003214, RZ Ctrl: 0.02138 ± 0.004657; P4 d3 hAFSC-EVs BZ: 0.02492 ± 0.004609, RZ: 0.03770 ± 0.007848; *n* = 8 mice per experimental group; P4 d3 Sham MI 0.01230 ± 0.004847, *n* = 4 mice; *p = 0.0477). Myo: myocardium; MI: myocardial infarction; LV: left ventricle.

**FIGURE 5:**
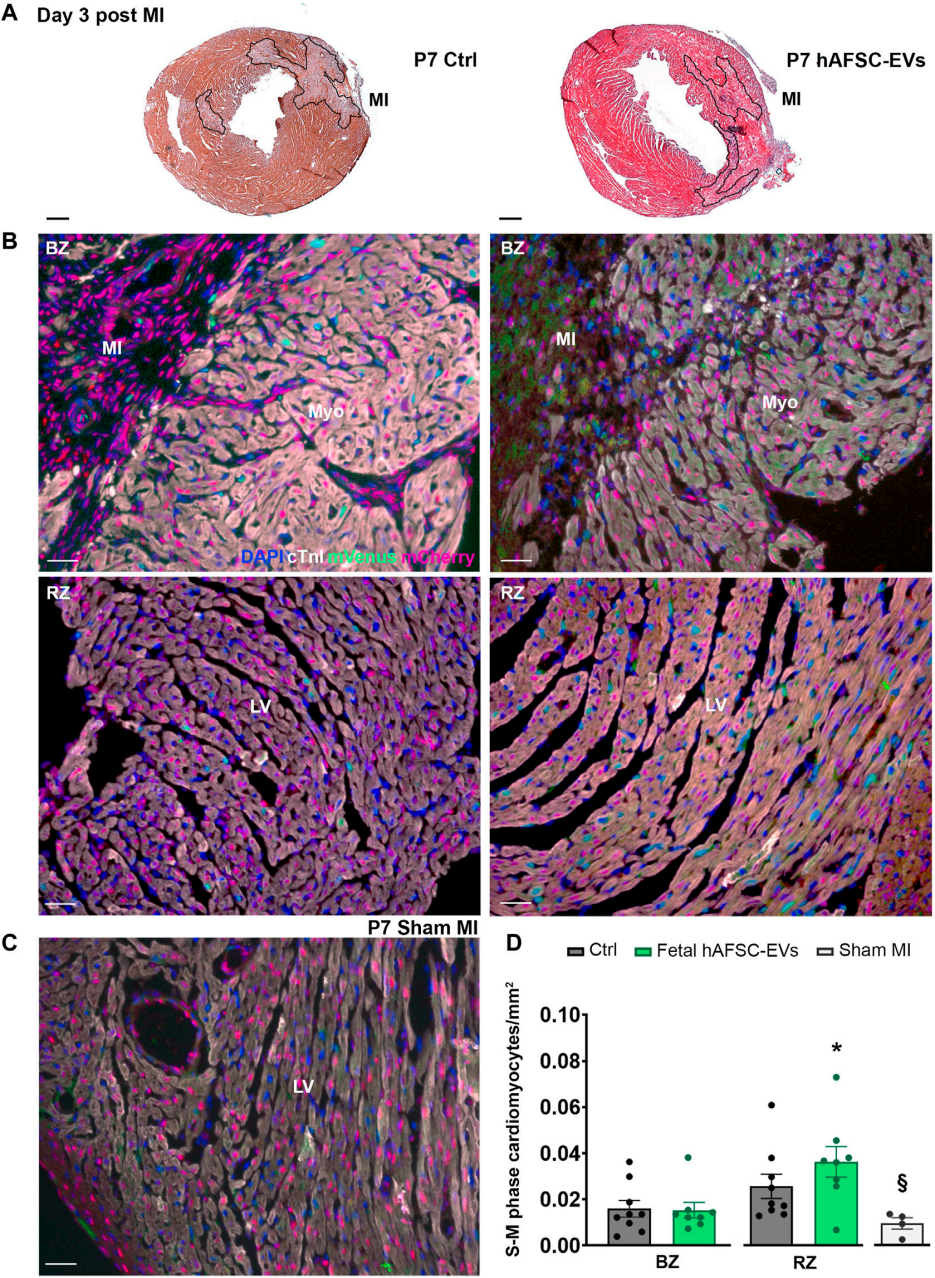
*In vivo* cardiogenic effect of fetal hAFSC-EVs on P7 neonatal mouse heart at 3 days post MI. **(A)** Representative pictures of hematoxylin and eosin staining on cryo-sections of R26pFUCCI2^+/−^ 7-day-old (P7) mouse myocardial tissue in: vehicle-treated control condition (PBS solution, left panel, Ctrl) and following intraperitoneal injection of 4.5 μg fetal hAFSC-EVs (hAFSC-EVs, right panel); myocardial infarct area (MI) is traced in black, scale bar: 300 µm. **(B)** Representative pictures of immunostaining analysis on cryo-sections of R26pFUCCI2^+/−^ 7-day-old (P7) mouse myocardial tissue in: vehicle-treated control conditions (PBS solution, left panel, Ctrl), following intraperitoneal injection with 4.5 μg fetal hAFSC-EVs (hAFSC-EVs, right panel) or in Sham control conditions [**(C)**, Sham MI, for DAPI (blue), cardiac troponin I (cTnI, white), mVenus-Geminin (mVenus, green), and mCherry-Cdt1 (mCherry, red), at 3 days post MI (P7 d3), scale bar: 50 µm. **(D)** Evaluation of mVenus-positive and cTnI-positive resident cells (S-M phase cardiomyocytes/mm^2^) receiving hAFSC-EVs treatment compared to vehicle Ctrl solution in the MI border zone (BZ, upper panel) and in the remote zone (RZ, lower panel) of the left ventricle (LV). All values are expressed as mean ± s.e.m. of cTnI-positive cardiomyocytes expressing mVenus-positive nuclei per mm^2^ (P7 d3 Ctrl BZ: 0.01595 ± 0.003586, RZ: 0.02566 ± 0.005207, *n* = 9 mice; P7 d3 hAFSC-EVs BZ: 0.01529 ± 0.003393; RZ: 0.03629 ± 0.0.006650, *n* = 8 mice; P4 d3 Sham MI 0.009442 ± 0.002469, *n* = 4 mice; *p = 0.0312 hAFSC-EVs RZ versus Ctrl BZ and *p = 0.0304 hAFSC-EVs RZ versus hAFSC-EVs BZ; ^§^ *p* = 0.0218 Sham MI versus hAFSC-EVs RZ). Myo: myocardium; MI: myocardial infarction; LV: left ventricle.

MI injury did not represent a trigger sufficient to induce resident cardiomyocytes to initiate their renewal program at either P4 or P7 stages as compared to the sham-operated (Sham MI) control group (Figures 4, 5, and Supplementary Figures S5, S6).

While intraperitoneal administration of fetal hAFSC-EVs soon after MI did not exert any effect on resident cardiomyocytes within the left ventricle (LV) close to the infarct area (namely the border zone, BZ, Figure 4), a 1.8-fold (*p < 0.05) increase in cardiomyocyte cell cycle progression was appreciated within the viable myocardium (the LV remote zone, RZ) following hAFSC-EVs treatment over Ctrl vehicle solution administration, at 3 days from injury (Figure 4). When investigating the fetal hAFSC-EV regenerative effect at later stage, 7 days after MI in the P4 mouse pups, no difference was appreciated between pups receiving hAFSC-EVs over the Ctrl group in either the RZ or the BZ areas (Supplementary Figure S5). Yet, hAFSC-EV stimulatory effect in sustaining cardiomyocyte cell cycle progression in the remote myocardium was detected within a week over sham-operated control group (^§^ *p* < 0.05, Supplementary Figure S5).

Likewise, the amount of mVenus-positive endogenous cardiomyocytes in the P7 myocardium was comparable between the Ctrl and the fetal hAFSC-EV–treated group in the injury BZ. Resident cardiomyocyte cell cycle progression in the RZ of P7 mouse pups receiving hAFSC-EVs showed a mild positive trend compared to the corresponding area in the Ctrl group 3 days post MI, while being upregulated by 2-fold (*p < 0.05) over the most unresponsive part of the injured myocardial tissue, the border zone (Figure 5). hAFSC-EV–induced myocardial response in the short term after MI was also found significantly improved, when compared to sham-operated controls (^§^ *p* < 0.05, Figure 5). Yet, this paracrine effect was reduced within a week from injury and treatment, with no significant improvement in resident cardiomyocyte cell cycle re-entry following treatment (Supplementary Figure S6).

### 3.4 Fetal hAFSC-EVs Are Enriched With Agrin as Potential Molecular Candidate of Action

Extracellular vesicles separated and concentrated from hAFSC-CM confirmed the canonical small EV phenotype, with expression of the multivesicular body biogenesis protein TSG101 and Syntenin-1, as reference markers (Kugeratski et al., 2021), while lacking the endoplasmic reticulum antigen GRP94 and showing heterogenous distribution of exosomal tetraspanins over their size range, with enriched expression of CD81 in both small (100–160 nm size, ****p < 0.0001) and large EVs (160–900 nm size, **p < 0.01, ****p < 0.0001) and CD63 in small EVs (100–160 nm size, ^#^ *p* < 0.05, Supplementary Figures S4B,C).

**FIGURE 6:**
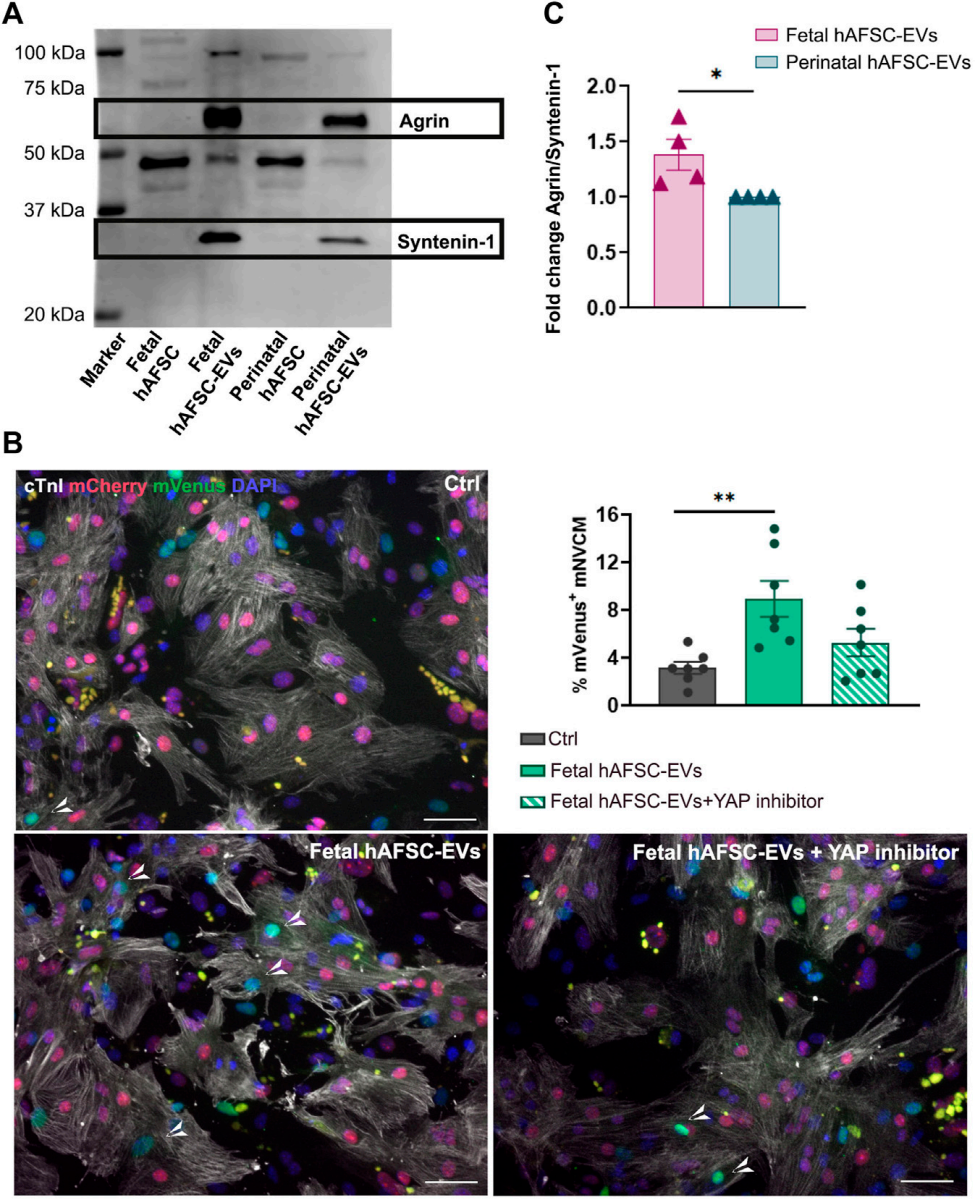
Agrin as molecular candidate of hAFSC-EV effect on mNVCM. **(A)** Left panel: representative Western blot of Agrin expression within fetal hAFSC-EVs over perinatal hAFSC-EVs, normalized to the presence of the small EV marker Syntenin-1; right panel: densitometry analysis of protein band signal by Agrin over Syntenin-1–fold change expression as mean ± s.e.m. (Fold Change Agrin/Syntenin-1) of *n* = 4 independent experiments (fetal hAFSC-EVs: 1.38 ± 0.14; perinatal hAFSC-EVs as calibrator: 1; *p = 0.0348). **(B)** Representative images of R26pFUCCI2^+/−^ mNVCM following treatment with fetal hAFSC-EVs with or without YAP pathway inhibitor (10 μM Verterporfin solution) or in vehicle-treated control condition (PBS solution, Ctrl) by immunostaining for cardiac troponin I (cTnI, white); mCherry-Cdt1 (mCherry, red), mVenus-Geminin (mVenus, green) and DAPI (blue), scale bar: 50 µm. **(C)** Percentage of mVenus-positive and cTnI-positive cells (mVenus^+^ mNVCM) stimulated with fetal hAFSC-EVs alone or with YAP inhibitor (fetal hAFSC-EVs + YAP inhibitor) compared to vehicle-treated cells (PBS solution, Ctrl). All values are expressed as mean ± s.e.m. percentage of mVenus^+^ mNVCM (% mVenus^+^ mNVCM) of *n* = 7 independent experiments (Ctrl: 3.14 ± 0.50%; fetal hAFSC-EVs: 8.92 ± 1.50%; fetal hAFSC-EVs + YAP inhibitor: 5.27 ± 1.15%; **p = 0.0054).

We previously characterized the proteomic cargo of both fetal and perinatal hAFSC-EVs, with indication of different enrichment of the first ones over the latter for some extracellular matrix components, including the proteoglycan Agrin (Costa et al., 2021). From our immunostaining analyses on treated mNVCM, fetal hAFSC-EVs demonstrated to be the most cardioactive secretome formulation in stimulating cardiomyocyte cell cycle activity. Here we also confirmed the presence of a short Agrin isoform (less than 75 kDa), as significantly enriched (****p < 0.0001) within fetal hAFSC-EVs over perinatal ones by about an additional 40% (Figure 6A). Agrin has been described as important mediator for heart regeneration in neonatal mice as inducing nuclear YAP translocation, thus promoting the activation of cardiomyocyte renewal (Bassat et al., 2017). Therefore, to validate a putative mechanism of action of the most cardioactive fetal hAFSC-EVs *via* Agrin–YAP signaling axis, Verteporfin, a specific YAP pathway inhibitor, was used on mNVCM stimulated *in vitro*. Cell cycle progression traced by nuclear mVenus expression in R26FUCCI2^+/−^ mNVCM (*n* = 7) was analyzed under the following treatment: vehicle control solution (1X PBS, Ctrl), fetal hAFSC-EVs alone (Fetal hAFSC-EVs) and fetal hAFSC-EVs with addition of YAP inhibitor (Fetal hAFSC-EVs + YAP inhibitor). Fetal hAFSC-EV stimulation increased the percentage of mVenus^+^ mNVCM by about 2.84-fold over the Ctrl group (**p < 0.01). The presence of the YAP inhibitor during hAFSC-EV treatment resulted in a decreasing trend of cardiogenic effect by about 40%, suggesting a possible, yet mild, putative involvement of YAP signaling in the hAFSC-EV paracrine mechanism (Figure 6B).

## 4 Discussion

This study shows that immature fetal hAFSC obtained from leftover samples of II trimester prenatal diagnosis amniocentesis secrete extracellular vesicles as their most cardioactive secretome formulation to support cell cycle re-entry and proliferation of murine cardiomyocytes. We have recently reported that fetal and perinatal hAFSC share a comparable phenotype and secretory efficiency, with a different profile in the paracrine cargo of their secretome formulations, according to the gestational stage of cell isolation (Costa et al., 2021). Here we further confirm that the secretome formulation of more immature cells are endowed with enhanced cardiogenic paracrine potential over their corresponding perinatal counterpart, pointing at fetal hAFSC as candidate cell source for future translational studies.

We previously demonstrated that both the whole fetal hAFSC secretome (hAFSC-CM) and the extracellular vesicles derived from it (hAFSC-EVs) can sustain cardiomyocyte cell cycle re-entry up to the S phase by bromodeoxyuridine (BrdU) uptake, in a preclinical mouse model of myocardial infarction (MI), extending such effect to up of 28 days after single intramyocardial injection (Balbi et al., 2019a). While such encouraging results suggested some degree of regenerative potential in the fetal hAFSC secretome, BrdU uptake presents some concerns, as merely indicating integration within DNA and thus tracing cell engagement to S phase, without providing any information regarding further true commitment to G2/M phase. To overcome such limit, here we employed a specific transgenic mouse model, known as R26pFUCCI2 mouse, described for the first time by (Abe et al., 2013). FUCCI labeling technology represents a useful tool to investigate cardiomyocyte cell cycle dynamics by means of mutually exclusive fluorescent nuclear signals, as recently reported by others as well (Alvarez et al., 2019; Baniol et al., 2021; Del Campo et al., 2022). The heterozygous R26pFUCCI2 mouse model used in our study is endowed with constitutive expression of a transgene probe system targeting the cell cycle–regulating protein, Cdt1 and its inhibitor Geminin, which are implicated in the DNA replication system (Wohlschlegel et al., 2000). The expression levels of these two proteins are inversely proportional and they change during cell cycle progression. Cdt1 levels are the highest in G1 phase and they decrease after the S phase. In contrast, Geminin levels peak during S and G2 phase, but drop drastically during late mitosis and G1 phase. Thus, R26pFUCCI2 system allows the fluorescent labeling of cell cycle G1 phase by expression of nuclear mCherry-Cdt1 and S/G2/M phases by expression of nuclear mVenus-Geminin (Abe et al., 2013).

Notably, while the whole fetal hAFSC secretome—as the cell-conditioned medium, hAFSC-CM—showed promising trend in the increase of responsive R26pFUCCI2 neonatal cardiomyocytes re-entering cell cycle advancement, only fetal hAFSC-EVs concentrated and separated from it significantly enhanced cell progression over G1 phase in culture. Notably, we have previously demonstrated the different cardiovascular potential of hAFSC-CM versus hAFSC-EVs in the adult mouse model of acute MI, where total hAFSC secretome exerted distinct pro-angiogenic effects that the EV counterpart was not able to evoke in the injured myocardium; *viceversa*, hAFSC-EVs showed enhanced cardioprotective rescue capacity in supporting cardiac function in the long-term (Balbi et al., 2019a). Since the total amount of particles within the hAFSC-CM and hAFSC-EV formulations was not statistically different, the different cardiogenic effect we appreciated in this study, may be related to the modulatory activity of soluble factors other than the EVs which are present in the hAFSC-CM. Nuclear co-expression of the chromosomal passenger enzyme Aurora B kinase further indicated that most of stimulated cardiomyocytes were actually proceeding over S and G2 phases into mitotic prophase (Adams et al., 2001; Murata-Hori et al., 2002). Moreover, fetal hAFSC-EVs demonstrated to actively trigger true cardiomyocyte division, as shown by Aurora B kinase expression in the cell midbody structure, thus indicating cytokinesis being in progress. hAFSC secretome stimulation also induced the expression of an altered structural phenotype in the cardiomyocytes treated *in vitro*, with sign of sarcomeric disassembly by disorganized expression of cardiac alpha-actinin, suggesting cardiomyocyte de-differentiation, a phenotypic feature required for cell cycle re-entry and renewal. Fetal hAFSC-EVs targeted the actin cytoskeleton architecture by significantly decreasing *Cofilin-2* gene expression while mildly sustaining F-actin polymerization, as consequence. Indeed, Cofilin-2 represents a key regulator of cardiomyocyte cell cycle re-entry by modulating actin polymerization and antagonizing actin polymerization into F filaments, thus increasing the G-Actin pool within the cytoplasm; in particular, it has been previously described that miR-199-a-3p targets *Cofilin-2* so to induce sarcomere disassembly and trigger cardiomyocyte proliferation (Torrini et al., 2019). Notably, additional bioinformatics analyses on the RNA content within the fetal hAFSC-EV cargo previously reported in (Costa et al., 2021) indicated that the 100 most enriched miRNA in the EVs, including miR-199-a-3, preferentially target the *Cofilin-2* gene.

The early postnatal murine heart is endowed with significant regenerative activity following critical injury, such as MI, by means of efficient resident cardiomyocyte renewal and division. Nonetheless, this regenerative program is active only within a very narrow postnatal window, with complete loss of effect after the first week from birth (P7). The 7-day-old (P7) mouse pup represent the limit of the neonatal cardiac regenerative window, being more similar to the adult heart in activating defective cardiac repair over restorative myocardial renewal (Porrello et al., 2013; Aurora et al., 2014; Lam and Sadek, 2018). In order to validate *in vivo* the results obtained on neonatal cardiomyocytes cultured *in vitro*, the cardiogenic potential of fetal hAFSC-EVs to unlock and extend such limit was assessed in a preclinical model of myocardial infarction in both P4 (i.e., representing half-way the decrease of regenerative potential) and P7 mouse pups (indicating the termination of the postnatal regenerative window). Fetal hAFSC-EVs induced an increase in resident cardiomyocyte cell cycle activity within the short-term following injury (i.e., 3 days after MI in P4 and P7 murine heart). Notably, cardiomyocyte cell cycle progression was appreciated in the left ventricle myocardium not closely related to the injured area, as suggesting that the developmental renewal program was reinstated in those viable cells organizing the regenerative advancing front as during zebrafish heart regeneration (Kikuchi et al., 2010; Yin et al., 2012). Fetal hAFSC-EV cardiogenic effect was blunted after 7 days from injury. We cannot exclude that such transient cardiogenic effect may be influenced by technical limitations in the hAFSC-EV delivery route and in the lack of follow-up treatments over time, to sustain their cardioactive influence. Here we chose to deliver fetal hAFSC-EVs *via* intraperitoneal (i.p.) administration, over more invasive intramyocardial injection, in compliance with the 3R’s principle.

As a matter of fact, different routes have been investigated in order to deliver mesenchymal stromal cell–EVs (MSC-EVs) in several preclinical model of disease, including intravenous administration, and local and intraperitoneal injections, as comprehensively reviewed in (Akbari et al., 2020). Intraperitoneal administration in particular has been applied in some recent studies investigating the regenerative activity of MSC-EVs in newborn rodent preclinical models. EVs obtained from rat bone marrow- and human umbilical cord MSC have been administered daily by i.p. injections in neonatal rats developing bronchopulmonary dysplasia early after birth and counteracted disruption of alveolarization, promoted angiogenesis and ameliorated pulmonary hypertension and right ventricular hypertrophy (Braun et al., 2018; Chaubey et al., 2018). Likewise, i.p. delivery of human umbilical cord MSC EVs exerted beneficial effects by preserving cochlear hair cells in mice receiving chronic cisplatin injection as preclinical model of ototoxicity-induced hearing loss (Tsai et al., 2021). Furthermore, i.p. injection of murine adipose tissue MSC-EVs in a preclinical model of inflammatory bowel disease (IBD) in mice with dextran sulfate sodium-induced acute colitis decreased colon injury and reduced TNF-α expression, while increasing Treg cells and TGF-β and IL-10 levels in the spleen and lymph nodes (Heidari et al., 2021). While i.p. delivery is generally well-tolerated in rodent preclinical models of disease, thus supporting overall survival and recovery, it may also present specific drawbacks. Indeed, hAFSC-EVs may struggle to reach the injured myocardial tissue, due to off-target biodistribution in other tissues, with unspecific or delayed outcome. As a matter of fact, i.p. delivery has been shown to be remarkably effective when targeting organs associated with the peritoneum cavity. On the other side, systemic delivery *via* intravenous (i.v.) infusion may provide a more clinically compliant option for follow-up treatments, offering prompt systemic distribution and improving the chances of cardiac homing; however, this approach does not prevent possible side effects either. Moreover, small EVs delivered systemically *via* i.v. administration have demonstrated limited half-life in the mouse circulation, up to very few minutes (Takahashi et al., 2013).

Another relevant aspect to be acknowledged about our *in vivo* delivery protocol is that we could not provide follow-up treatment. The main reason was to avoid any additional stress in the mother caused by frequent manipulation of the treated pups, thus prompting her to neglect them. Likewise, although progenitor-derived EVs have been broadly described as immunological inert and immunomodulatory agents, we cannot then exclude that repeated i.p. treatments may increase the risk of local sensitization leading to *in situ* activation of immune cells.

When fetal hAFSC-EVs were administered by intramyocardial delivery soon after MI, we reported long-lasting beneficial effects within the adult cardiac tissue with increased BrDU uptake in resident cardiomyocyte (Balbi et al., 2019a). Indeed, the relevance of local delivery in supporting paracrine stimulation on resident cardiomyocyte renewal seems to be highlighted by independent work as well, in which epicardial-derived progenitor cell–EVs were able to maintain a proliferative response *in vivo* for a bit longer, during and beyond the P7 murine neonatal window, following their direct intracardiac injection during MI, despite this limiting the sample size (Del Campo et al., 2022). While direct and localized injection may increase the efficiency of local targeting, this approach is likely quite invasive and hardly clinically feasible, especially for chronic cardiac disease and heart failure. Therefore, suitable alternatives should be optimized in order to define a paracrine therapy protocol ensuring therapeutic efficacy together with great potential for translational medicine and their successful application in the clinical scenario. In light of these considerations, customized engineering of the hAFSC-EV surface for the specific targeting of cardiomyocytes and the injured myocardium may represent a desirable strategy. This should be further combined with the design of *ad hoc* biocompatible hydrogel formulations for prolonged and controlled EV release *in vivo*, in order to increase stability, retention and to overcome the need of multiple follow-up administrations in the long term.

In our previous study assessing the cardioprotective role of hAFSC-EVs in the preclinical adult mouse model of MI, we demonstrated that a single intramyocardial administration of EVs resulted in the significant rescue of cardiac function in the long-term, without any collateral effects (Balbi et al., 2019a). We appreciated that it would have been relevant to evaluate whether hAFSC-EVs could exert the same cardioactive paracrine influence on the neonatal injured myocardium as well; nonetheless, cardiac function imaging on such juvenile MI model may represent a technical challenging task in our setting.

The regenerative competence of stem- and progenitor cell–EVs is acquired from their cargo of paracrine mediators, which are sorted in the vesicle by the parental secreting cell as mirroring its potential. From the earlier molecular profiling we have performed on fetal versus perinatal hAFSC secretome formulations, we revealed that they shared a stable signature group of miRNAs (Costa et al., 2021). Nevertheless, additional bioinformatic analyses performed here indicated that immature fetal hAFSC-EVs may harbor a more cardiogenic regulatory RNA content targeting *Cofilin-2* and cytoskeleton re-organization. While we previously appreciated that perinatal hAFSC-EVs can deliver soluble factors involved in endothelial cell migration, immune-modulation and neurotrophic potential stimulation (Costa et al., 2021), here we confirmed that fetal hAFSC-EVs, as the most cardiogenic secretome formulation, are significantly enriched with a short isoform of the extracellular matrix proteoglycan Agrin. Indeed, Agrin has been shown to be a master regulator during neonatal heart regeneration in mice, as acting *via* the YAP signaling axis and promoting murine and human cardiomyocyte cell division (Bassat et al., 2017). In order to assess whether Agrin may be a candidate molecular effector underlying the cardioactive paracrine potential of fetal hAFSC-EVs, functional *in vitro* analyses were performed. Stimulating primary neonatal cardiomyocytes with fetal hAFSC-EVs in combination with the YAP inhibitor Verteporfin (Bassat et al., 2017) resulted in a negative trend of their cell cycle re-entry. Although we noticed some degree of inhibition in the hAFSC-EV cardiogenic paracrine effect, we may have to consider that some additional therapeutic mechanisms may be involved, such as a combinatorial effect involving Agrin and miRNAs within the EV cargo. Moreover, since the Agrin isoform detected on hAFSC-EVs is a shorter fragment of such protein, which may sound consistent with a 72 kDa cell-secreted variant similarly reported by others (Rivera et al., 2018), we cannot exclude that this isoform may not specifically or exclusively act on the target cardiomyocytes *via* YAP signaling. Therefore, additional detailed analyses on stimulated cardiomyocytes are necessary to pinpoint the specific molecular mechanism of action of fetal hAFSC-EVs, in order to enhance it. Further development will require comprehensive characterization of the ratio between hAFSC-EV functional activity and their appropriate dosing, as well as specific potency assays. Another limit we may acknowledge refers to the *in vivo* delivery system, which needs to be optimized in order to prolong and strengthen the transient cardiogenic potential of hAFSC-EVs so to ensure long-lasting and myocardial-specific results, while at the same time avoiding off-target systemic effects. This could be achieved by either engineering hAFSC-EV surface to implement their specific tropism toward cardiomyocytes or by developing biocompatible releasing systems to ensure hAFSC-EVs *in vivo* prolonged administration.

## 5 Conclusion

Collectively, our data suggests that fetal hAFSC can produce small EVs endowed with relevant cardiogenic effects to sustain myocardial renewal. Despite II trimester and III trimester hAFSC may present overlapping secretory efficiency, developmentally more immature fetal hAFSC-EVs have shown to be the more promising cardioactive fraction in supporting cardiomyocyte cell cycle re-entry. Yet, further functional analyses should be considered to better understand the molecular mechanism of action underlying the paracrine capacity of fetal hAFSC-EVs, in order to strengthen their potential and explore them as targeted delivery therapeutics for cardiac regenerative medicine. From a translational perspective, this study suggests that while fetal hAFSC-EVs may retain some degree of restorative capacity in supporting cardiomyocytes toward progression of the cell cycle up to true cell division, such effect may not therapeutically fulfill the expectations for adult patients experiencing myocardial infarction/cardiac dysfunction. Nevertheless, the fetal hAFSC-EV cardiogenic potential could be rather exploited in the neonatal scenario, where fetuses or newborns affected by congenital heart malformation with alteration of resident cardiomyocytes, may benefit of an innovative paracrine approach based on fetal hAFSC-EV prompt administration at/soon after birth or *in utero*. Such strategy may pave the way to promote myocardial tissue restoration by means of cell-free, EV-based advanced medicinal products, in combination with standard *in utero* or early postnatal reconstructive surgical interventions.

## Data Availability

Publicly available data sets were analyzed in this study. This data can be found at: Gene Expression Omnibus repository (www.ncbi.nlm.nih.gov/geo/) with accession number: GSE168152.
