# Supplementary figures and images for: Investigating the Paracrine Role of Perinatal Derivatives: Human Amniotic Fluid Stem Cell-Extracellular Vesicles Show Promising Transient Potential for Cardiomyocyte Renewal
Source: Front Bioeng Biotechnol. 2022 Jun 8;10:902038. doi: 10.3389/fbioe.2022.902038 (PMC9214211; doi:10.3389/fbioe.2022.902038)

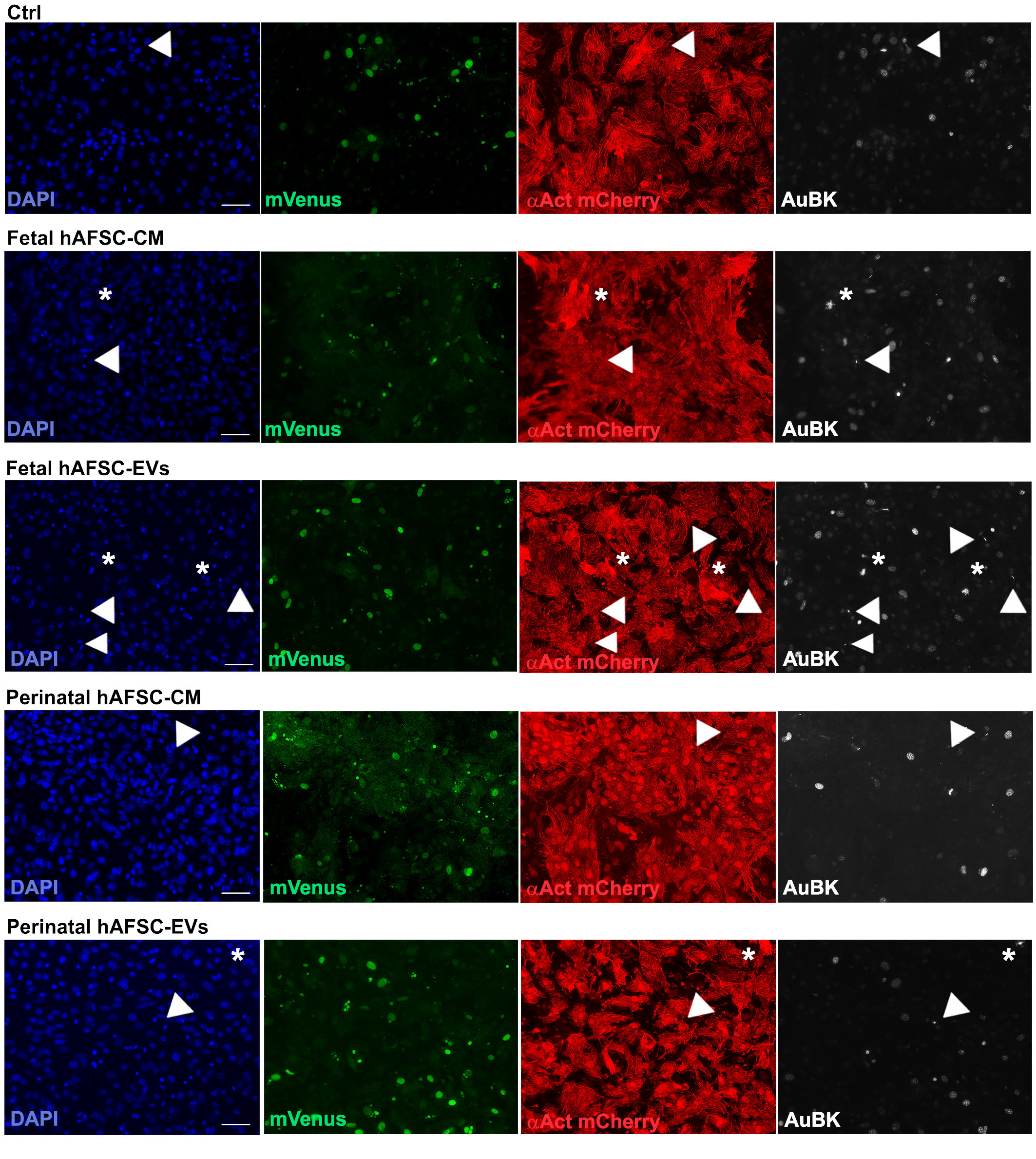

Supplement: Supplementary file 1 [file Image3.JPEG]

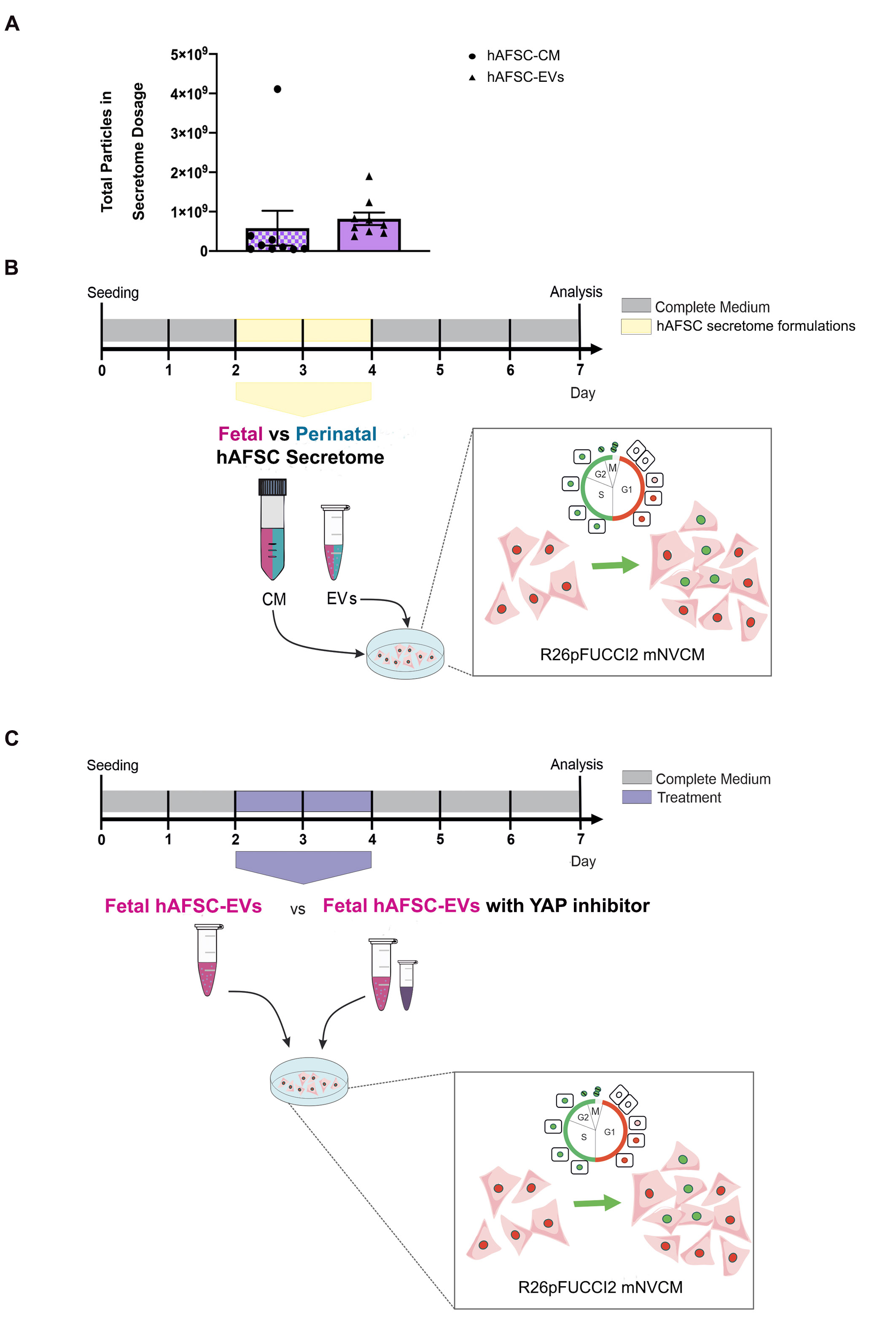

Supplement: Supplementary file 2 [file Image1.JPEG]

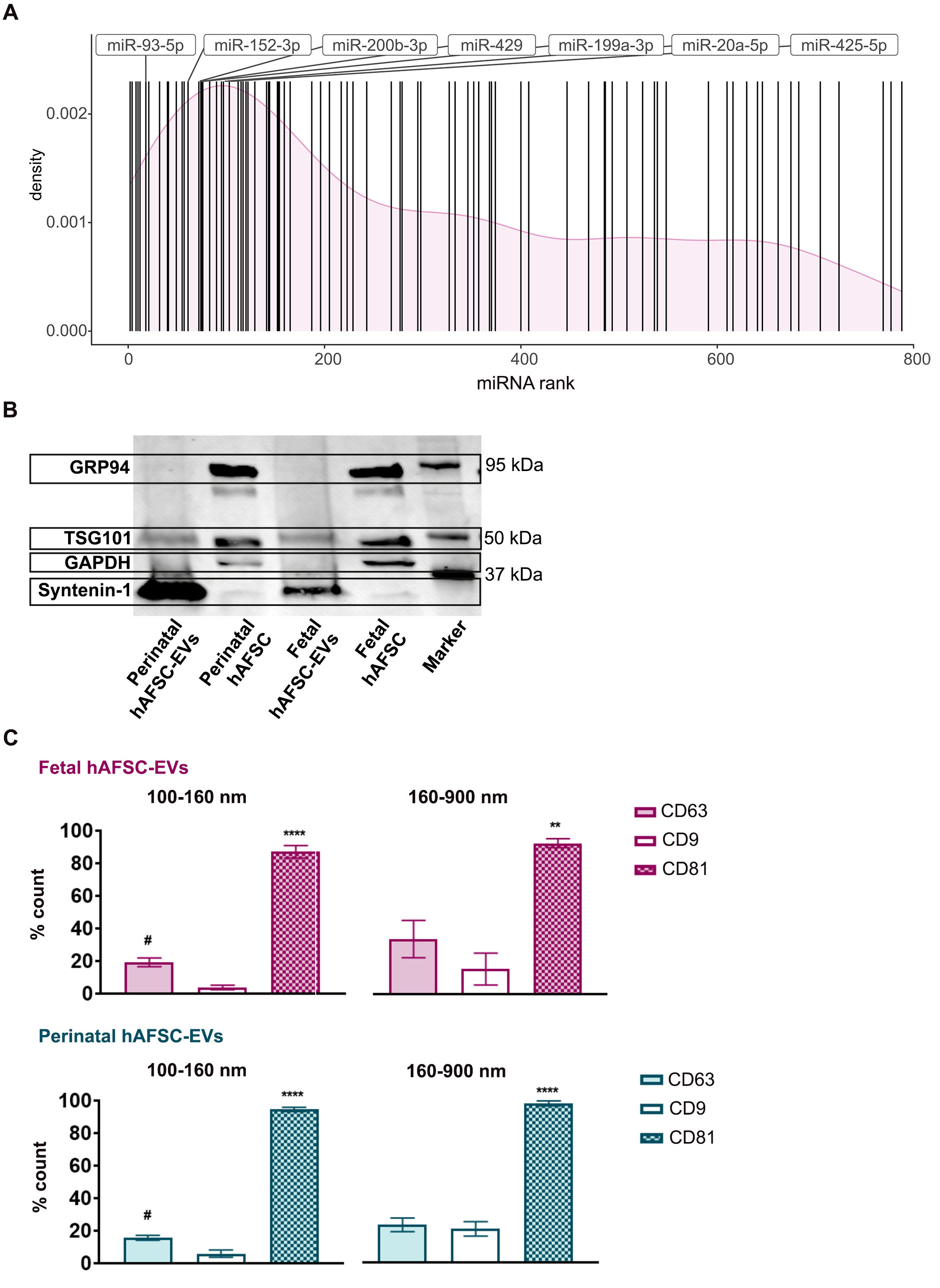

Supplement: Supplementary file 3 [file Image4.JPEG]

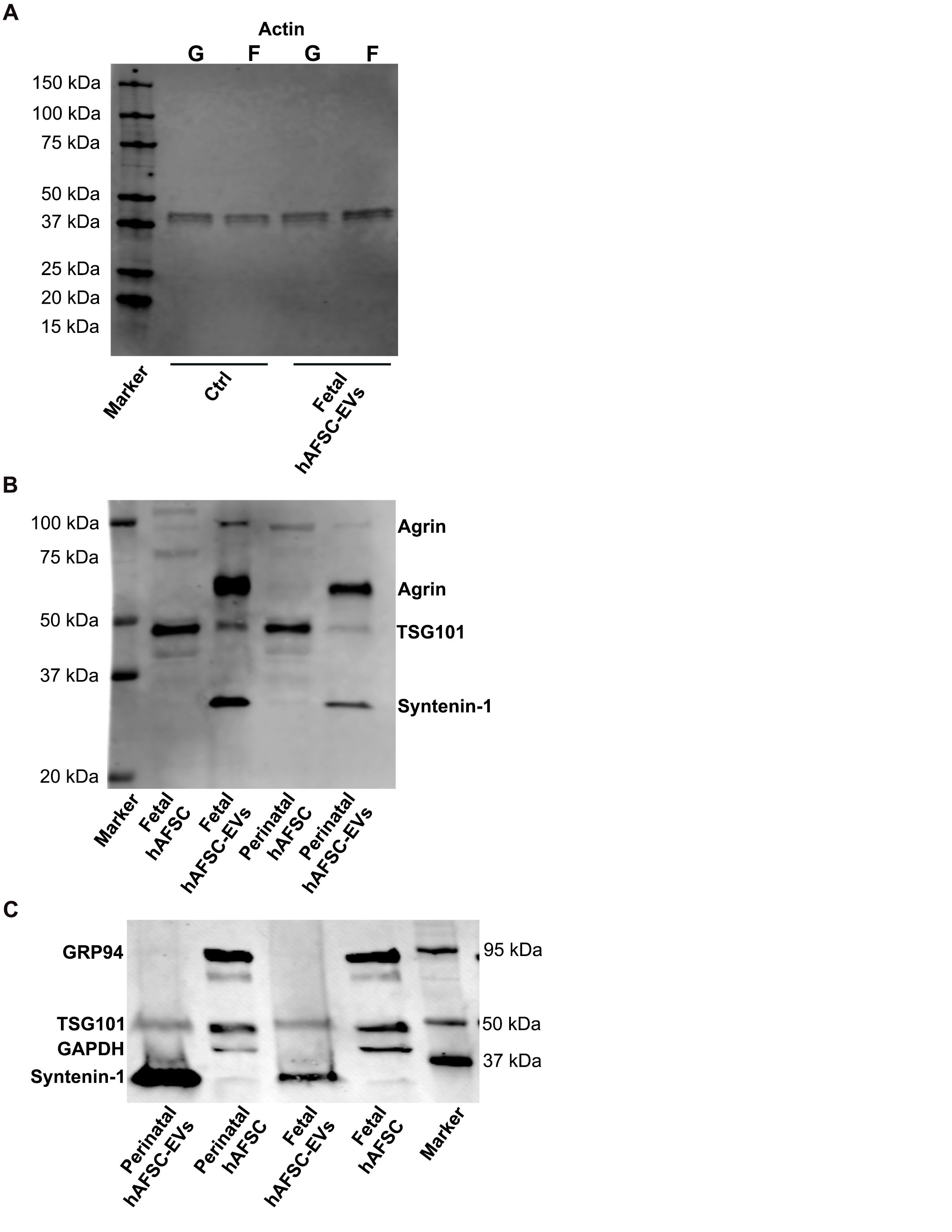

Supplement: Supplementary file 4 [file Image7.JPEG]

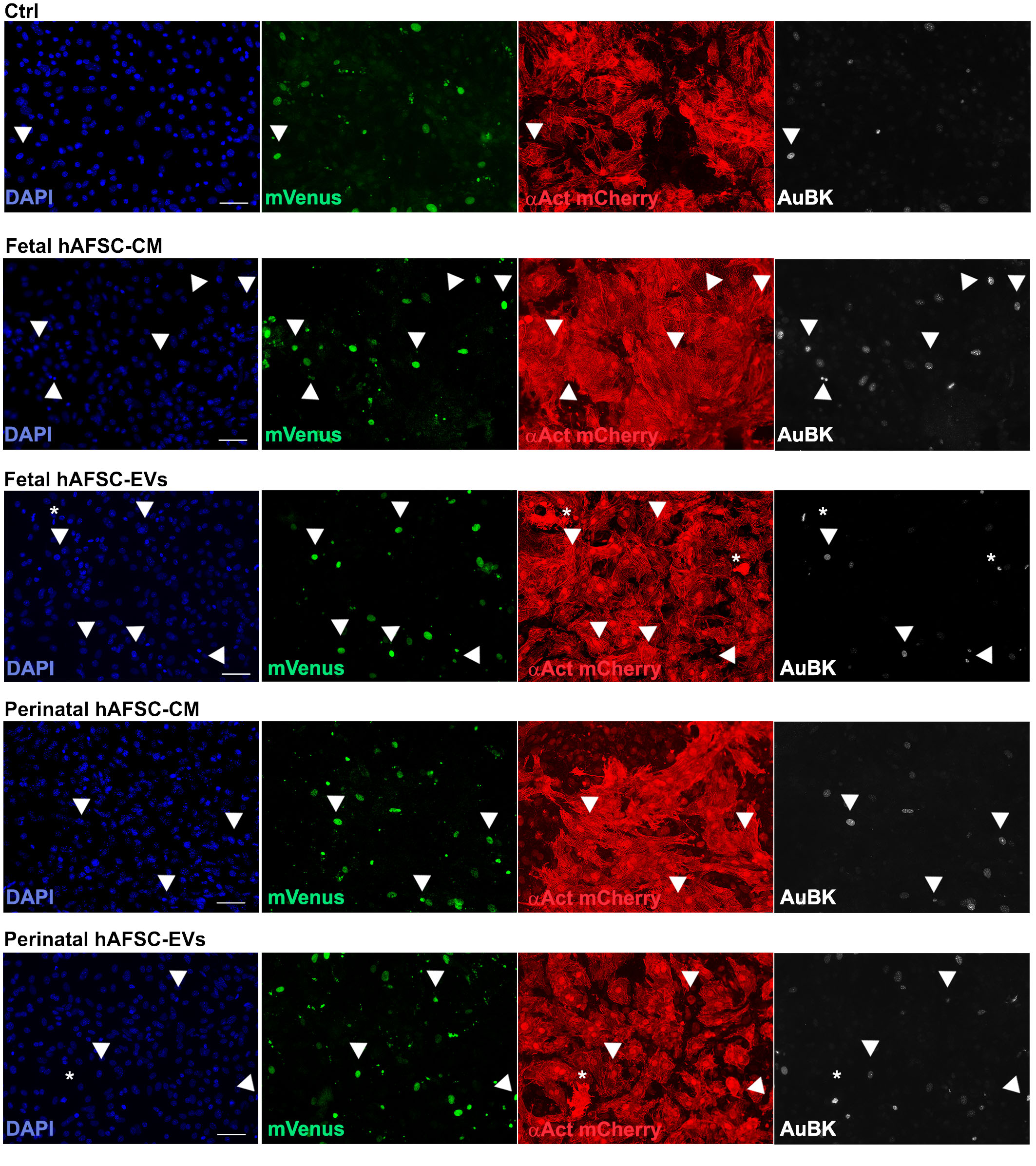

Supplement: Supplementary file 5 [file Image2.JPEG]

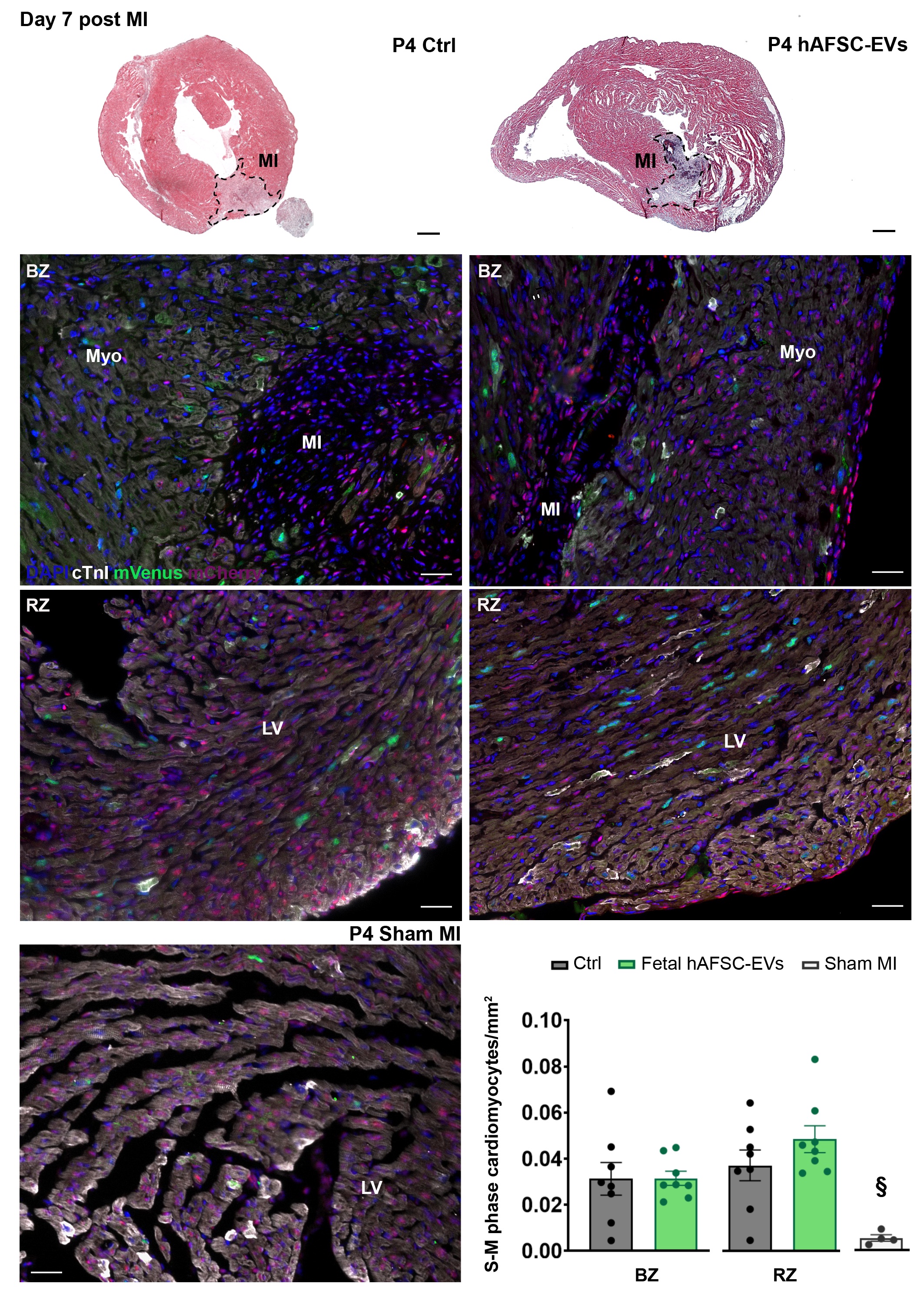

Supplement: Supplementary file 6 [file Image5.JPEG]

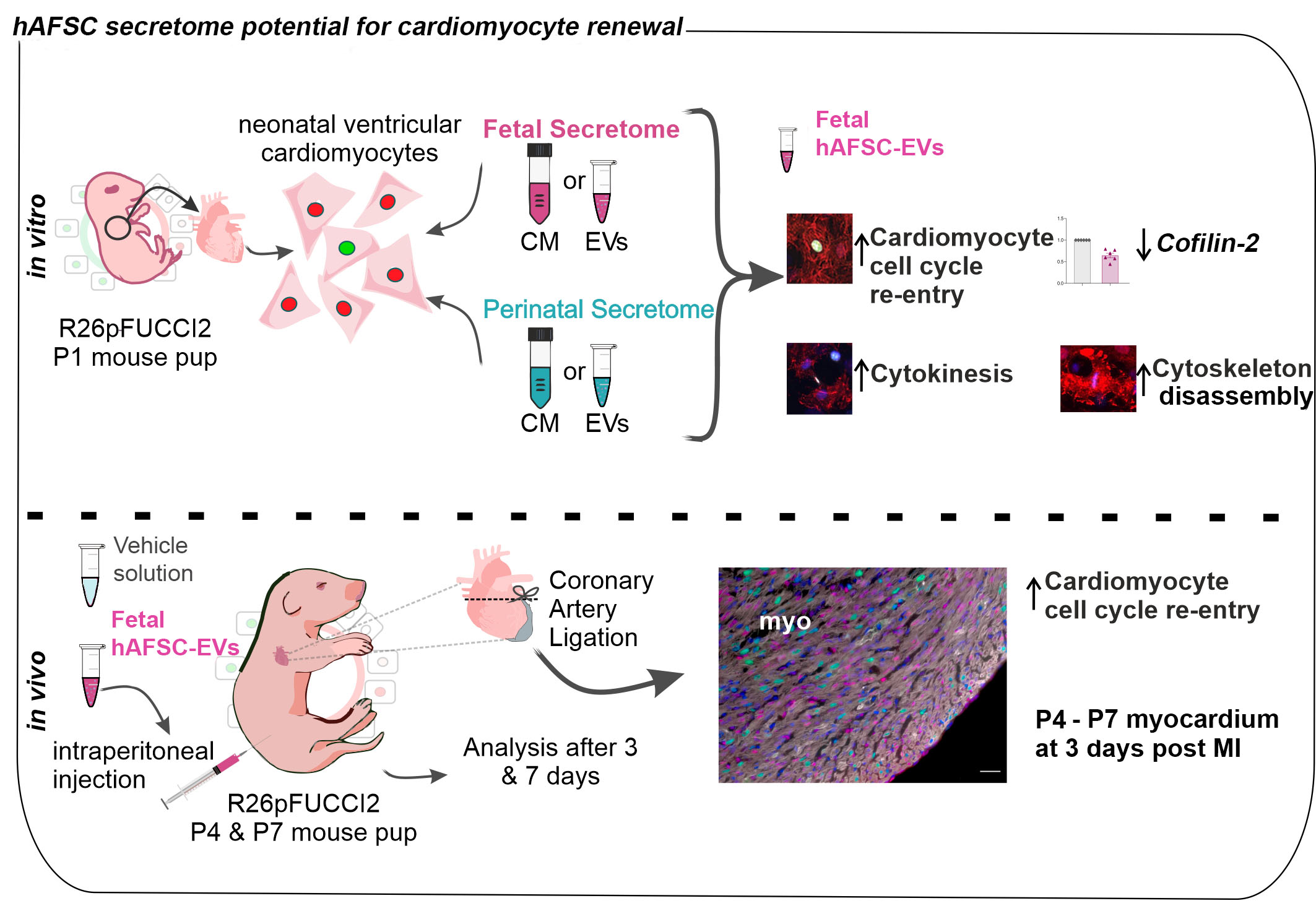

Supplement: Supplementary file 8 [file Image8.JPEG]

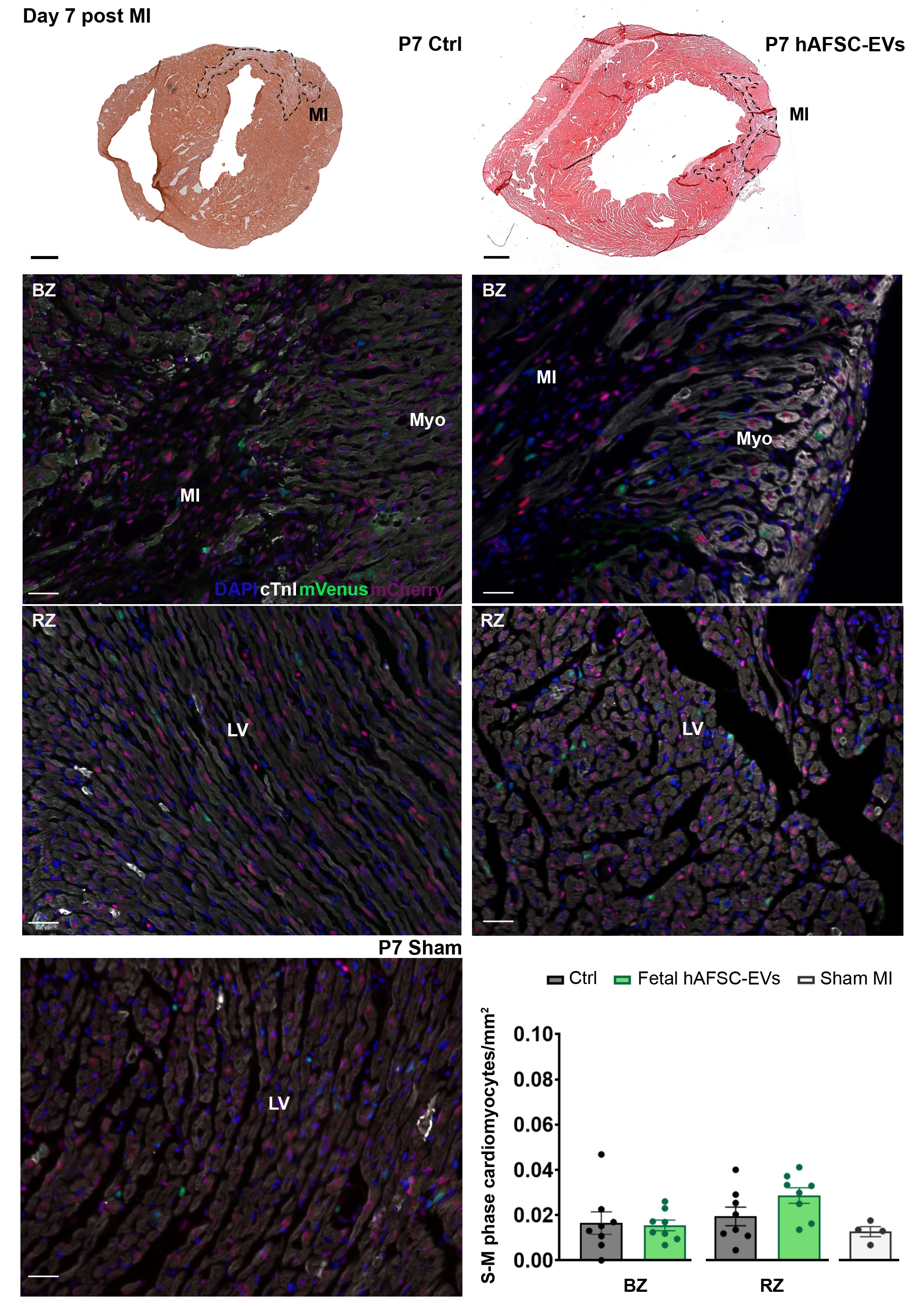

Supplement: Supplementary file 9 [file Image6.JPEG]
