# Supplementary material for: Investigating the Paracrine Role of Perinatal Derivatives: Human Amniotic Fluid Stem Cell-Extracellular Vesicles Show Promising Transient Potential for Cardiomyocyte Renewal
Source: Front Bioeng Biotechnol. 2022 Jun 8;10:902038. doi: 10.3389/fbioe.2022.902038 (PMC9214211; doi:10.3389/fbioe.2022.902038)
Supplement: Supplementary file 7 [file DataSheet1.pdf]

Supplementary Material

Investigating the paracrine role of perinatal derivatives: human amniotic fluid stem cell-extracellular vesicles show promising transient potential for cardiomyocyte renewal

Ambra Costa[1], Carolina Balbi[2,3,4], Patrizia Garbati[1], Maria Elisabetta Federica Palamà[1], Daniele Reverberi[5], Antonella De Palma[6], Rossana Rossi[6], Dario Paladini[7], Domenico Coviello[8], Pierangela De Biasio[9], Davide Ceresa[10], Paolo Malatesta[1,10], Pierluigi Mauri[6], Rodolfo Quarto[1,10], Chiara Gentili[1], Lucio Barile[4,11,12] and Sveva Bollini[1,*].

[1] Experimental Biology Unit, Department of Experimental Medicine (DIMES), University of Genova, Genova, Italy.

[2] Laboratory of Cellular and Molecular Cardiology, Istituto Cardiocentro Ticino, Ente Ospedaliero Cantonale, Lugano, Switzerland;

[3] Center for Molecular Cardiology, University of Zurich, Zurich, Switzerland.

[4] Laboratories for Translational Research, Ente Ospedaliero Cantonale, Bellinzona, Switzerland.

[5] Molecular Pathology Unit, IRCCS Ospedale Policlinico, San Martino, 16132 Genova, Italy.

[6] Proteomics and Metabolomics Unit, Institute for Biomedical Technologies (ITB-CNR), Segrate (MI), Italy.

[7] Fetal Medicine and Surgery Unit, IRCCS Istituto Giannina Gaslini, Genova, Italy.

[8] Laboratory of Human Genetics, IRCCS Istituto Giannina Gaslini, Genova, Italy.

[9] Prenatal Diagnosis Perinatal Medicine Unit, IRCCS Ospedale Policlinico San Martino, Genova, Italy.

[10] Cellular Oncology Unit, IRCCS Ospedale Policlinico San Martino, Genova, Italy.

[11] Laboratory for Cardiovascular Theranostics, Istituto Cardiocentro Ticino, Ente Ospedaliero Cantonale, Lugano, Switzerland.

[12] Faculty of Biomedical Sciences, Università Svizzera Italiana, Lugano, Switzerland.

Number of Figures: 7

Number of Tables: 0

Supplementary Figures

Supplementary Figure 1. Schematic of *in vitro* experimental design. (A) Evaluation of the amount of EV particles in the hAFSC-CM versus hAFSC-EV doses used to treat mNVCM *in vitro*, as for their concentration in micrograms (80 µg/ml corresponding to 16 µg versus 5 µg/ml corresponding to 1 µg per well, respectively) by means of nanoparticle tracking analysis (NTA). Total amount in the hAFSC-CM dose was $5.82 \times 10^8 \pm 4.43 \times 10^8$ particles; total amount in the hAFSC-EV dose was $8.19 \times 10^8 \pm 1.60 \times 10^8$ particles, $p=0.6218$; all values are expressed as mean ± s.e.m. of n=9 independent analyses. **(B)** *In vitro* experimental plan with hAFSC secretome formulations. **(C)** *In vitro* experiments with Yap inhibitor and hAFSC-EVs. Schematics have been produced using Smart - Servier Medical Art (https://smart.servier.com/).

Supplementary Figure 2. Details of *in vitro* immunostaining analyses on mNVCM following stimulation by fetal versus perinatal hAFSC secretome formulations. Representative images of R26pFUCCI2$^{+/-}$ mNVCM in vehicle treated control conditions (SF solution, Ctrl) or following treatment with fetal or perinatal hAFSC-CM or hAFSC-EVs. Each image refers to immunostaining analysis in Figure 1 for: DAPI (blue), mVenus-Geminin (mVenus, green), sarcomeric α-actinin (intracellular signal, αAct, red) and mCherry-Cdt1 (nuclear signal, mCherry, red), and Aurora B kinase (AuBK, white); scale bar: 50µm. White arrows point in each corresponding panel at: αAct-positive cells (mNVCM) with nuclear mVenus and AuBK signals relative to DAPI expression; white asterisks indicate mNVCM with disarranged α-actinin expression as sarcomeric disassembly feature.

Supplementary Figure 3. Details of *in vitro* immunostaining analyses on mNVCM with sign of cell division (cytokinesis) following fetal versus perinatal hAFSC secretome formulations. Representative images of R26pFUCCI2$^{+/-}$ mNVCM in vehicle treated control conditions (SF medium, Ctrl) or following treatment with fetal or perinatal hAFSC-CM or hAFSC-EVs. Each image refers to immunostaining analysis in Figure 2 for: DAPI (blue), mVenus-Geminin (mVenus, green), sarcomeric α-actinin (intracellular signal, αAct, red) and mCherry-Cdt1 (nuclear signal, mCherry, red), and Aurora B kinase (AuBK, white); scale bar: 50µm. White arrows indicate cells in each corresponding panel in which AuBK expression has been detected at cell midbody, corresponding to the completion of cytokinesis in αAct-positive cells (mNVCM); white asterisks indicate mNVCM with disarranged α-actinin expression as sarcomeric disassembly feature.

Supplementary Figure 4. hAFSC-EV characterization and bioinformatic analysis of the targets of their miRNA cargo. (A) Bioinformatic analysis of the 100 mostly enriched miRNAs within fetal hAFSC-EVs targeting CFL-2 gene. miRNAs were ranked basing on their relative enrichment levels (x axis) and the distribution of CFL-2 targeting miRNAs (y axis) was analyzed by density statistics. **(B)** Representative Western Blot of fetal- and perinatal hAFSC and their corresponding EVs for the expression of the exosomal multivesicular body biogenesis protein TSG101, the reference small EV marker Syntenin-1, the endoplasmic reticulum antigen GRP94 and the cell reference protein GAPDH. **(C)** CD63, CD9, CD81 tetraspanin antigen distribution and expression in fetal- and perinatal hAFSC-EVs by flow cytometry. The presence of tetraspanins was evaluated as mean percentage of single

antigen expression (% count), considering distribution within two size intervals: 100-160nm (for small and medium EVs) and 160-900nm (medium-large EVs). Upper panel: percentage of exosomal markers within fetal hAFSC-EVs; percentage value of CD63, CD9 and CD81 between 100- and 160nm: 19.24±2.69%; 3.72±1.36%; 87.09 ± 3.84%, respectively; $^{\#}$p=0.0189 CD63 versus CD9; ****p<0.0001 CD81 versus CD63 and ****p<0.0001 CD81 versus CD9. Percentage value of CD63, CD9 and CD81 between 160- and 900 nm: 34.31±11.63%; 15.56±9.96%; 93.95±2.82%, respectively; $^{\#}$ p=0.0175 CD63 versus CD9, ****p<0.0001 CD81 versus CD63 and ****p<0.0001 CD81 versus CD9. Lower panel: percentage of exosomal markers within perinatal hAFSC-EVs; percentage value of CD63, CD9 and CD81 between 100- and 160nm: 15.67±1.44%; 5.92±2.29%; 94.65±1.32%, respectively; **p=0.008 CD81 versus CD63 and **p=0.002 CD81 versus CD9. Percentage value of CD63, CD9 and CD81 between 160- and 900nm: 23.13±4.15%; 20.59±4.43%; 97.74±1.54%, respectively; ****p<0.0001 CD81 vs CD63 and ****p<0.0001 CD81 versus CD9. All values are expressed as mean ± s.e.m. of n=3 independent experiments. *kDa: kilo Dalton.*

Supplementary Figure 5. *In vivo* cardiogenic effect of fetal hAFSC-EVs on P4 neonatal mouse heart at 7 days post MI. Upper panel: representative pictures of hematoxylin and eosin staining on cryo-sections of R26pFUCCI2$^{+/-}$ 4-days-old (P4) mouse myocardial tissue in: vehicle-treated control condition (PBS solution, left panel, Ctrl) and following intraperitoneal injection of 4.5 μg fetal hAFSC-EVs (hAFSC-EVs, right panel); myocardial infarction (MI) area is indicated by black dashed line, scale bar: 300 μm. Lower panel: representative pictures of immunostaining analysis on cryo-sections of R26pFUCCI2$^{+/-}$ 4-days-old (P4) mouse myocardial tissue in: vehicle-treated control condition (PBS solution, left panel, Ctrl); following intraperitoneal injection with 4.5 μg fetal hAFSC-EVs (hAFSC-EVs, right panel) or in Sham control condition (bottom left image, Sham MI) for DAPI (blue), cardiac troponin I (cTnI, white), mVenus-Geminin (mVenus, green), and mCherry-Cdt1 (mCherry, red), at 7 days post MI (P4 d7), scale bar: 50μm. Evaluation of mVenus-positive and cTnI-positive resident cells (S-M phase cardiomyocytes/mm^2) with fetal hAFSC-EVs treatment over vehicle Ctrl solution in the MI border zone (BZ, upper panel) and in the remote zone (RZ, lower panel) of the left ventricle (LV). All values are expressed as mean ± s.e.m. of cTnI-positive cardiomyocytes expressing mVenus-positive nuclei per mm^2 (P4 d7 Ctrl BZ: 0.03126 ± 0.007062, RZ: 0.03717 ± 0.006666; P4 d7 hAFSC-EVs BZ: 0.03151 ± 0.0.003074, RZ: 0.04854 ± 0.005806; n=8 mice per experimental group; P4 d3 Sham MI 0.005327 ± 0.001420, n=4 mice), §p=0.0194 Sham MI versus Ctrl RZ and p=0.0009 Sham MI versus hAFSC-EVs RZ). *Myo: myocardium; MI: myocardial infarction; LV: left ventricle.*

Supplementary Figure 6. *In vivo* cardiogenic effect of fetal hAFSC-EVs on P7 neonatal mouse heart at 7 days post MI. Upper panel: representative pictures of hematoxylin and eosin staining on cryo-sections of R26pFUCCI2$^{+/-}$ 7-days-old (P7) mouse myocardial tissue in: vehicle-treated control condition (PBS solution, left panel, Ctrl) and following intraperitoneal injection of 4.5 μg fetal hAFSC-EVs (hAFSC-EVs, right panel); myocardial infarction (MI) area is indicated by black dashed line, scale bar: 300μm. Lower panel: representative pictures immunostaining analysis on cryo-sections of R26pFUCCI2$^{+/-}$7-days-old (P7) mouse myocardial tissue in: vehicle-treated control conditions (PBS solution, Ctrl, left panel), following intraperitoneal injection with fetal hAFSC-EVs (hAFSC-EVs, right panel) or in Sham control conditions (bottom left image, Sham MI), for DAPI (blue), cardiac troponin I (cTnI, white), mVenus-Geminin (mVenus, green), and mCherry-Cdt1 (mCherry, red), at 3 days post MI (P7 d3), scale bar: 50μm. Evaluation of mVenus-positive and cTnI-positive resident cells (S-M phase cardiomyocytes/mm^2) receiving hAFSC-EVs treatment compared to vehicle Ctrl solution in the MI border zone (BZ, upper panel) and in the remote zone (RZ, lower panel) of the left ventricle

(LV). All values are expressed as mean ± s.e.m. of cTnI-positive cardiomyocytes expressing mVenus-positive nuclei per mm^2 (P7 d7 Ctrl BZ: 0.01650 ± 0.004948, RZ: 0.01943 ± 0.004102; P7 d7 hAFSC-EVs BZ: 0.01545± 0.002343, RZ: 0.02865 ± 0.003451); n=8 mice per experimental group. P7d7 Sham MI 0.01271 ± 0.002234, n=4 mice). *Myo: myocardium; MI: myocardial infarction; LV: left ventricle.*

Supplementary Figure 7. Uncropped pictures of Western Blot images. (A) Uncropped image of representative Western Blot of F-Actin and G-Actin expression in mNVCM in vehicle control solution condition (Ctrl) and with treatment by fetal hAFSC-EVs. **(B)** Uncropped image of the representative Western Blot for the expression of Agrin, TSG101 and Syntein-1 in fetal- and perinatal hAFSC-EVs and in their corresponding secreting cells (perinatal and fetal hAFSC). **(C)** Uncropped image of the representative Western Blot of fetal- and perinatal hAFSC and hAFSC-EV characterization for the expression of GTP94, TSG101, GADPH and Syntenin-1; *kDa: kilo Dalton.*
